# PPP2R5C Couples Hepatic Glucose and Lipid Homeostasis

**DOI:** 10.1371/journal.pgen.1005561

**Published:** 2015-10-06

**Authors:** Yong-Sheng Cheng, Oksana Seibert, Nora Klöting, Arne Dietrich, Katrin Straßburger, Sonia Fernández-Veledo, Joan J. Vendrell, Antonio Zorzano, Matthias Blüher, Stephan Herzig, Mauricio Berriel Diaz, Aurelio A. Teleman

**Affiliations:** 1 German Cancer Research Center (DKFZ), Heidelberg, Germany; 2 Department of Medicine, University of Leipzig, Leipzig, Germany; 3 Hospital Universitari de Tarragona Joan XXIII, Institut d´Investigació Sanitària Pere Virgili. Universitat, Rovira i Virgili, CIBERDEM, Tarragona, Spain; 4 Hospital Universitari de Tarragona Joan XXIII. Institut d´Investigació Sanitària Pere Virgili Universitat Rovira i Virgili, CIBERDEM, Tarragona, Spain; 5 Institute for Research in Biomedicine (IRB Barcelona), Departament de Bioquímica i Biologia Molecular, Universitat de Barcelona, and CIBERDEM, Barcelona, , Spain; 6 Institute for Diabetes and Cancer, Helmholtz Center Munich, Neuherberg, Germany, and Joint Heidelberg-IDC Translational Diabetes Program, University Hospital Heidelberg, Heidelberg, Germany; 7 Chair Molecular Metabolic Control, Medical Faculty, Technical University Munich, Germany; UCSF Diabetes Center, UNITED STATES

## Abstract

In mammals, the liver plays a central role in maintaining carbohydrate and lipid homeostasis by acting both as a major source and a major sink of glucose and lipids. In particular, when dietary carbohydrates are in excess, the liver converts them to lipids via de novo lipogenesis. The molecular checkpoints regulating the balance between carbohydrate and lipid homeostasis, however, are not fully understood. Here we identify PPP2R5C, a regulatory subunit of PP2A, as a novel modulator of liver metabolism in postprandial physiology. Inactivation of PPP2R5C in isolated hepatocytes leads to increased glucose uptake and increased de novo lipogenesis. These phenotypes are reiterated in vivo, where hepatocyte specific PPP2R5C knockdown yields mice with improved systemic glucose tolerance and insulin sensitivity, but elevated circulating triglyceride levels. We show that modulation of PPP2R5C levels leads to alterations in AMPK and SREBP-1 activity. We find that hepatic levels of PPP2R5C are elevated in human diabetic patients, and correlate with obesity and insulin resistance in these subjects. In sum, our data suggest that hepatic PPP2R5C represents an important factor in the functional wiring of energy metabolism and the maintenance of a metabolically healthy state.

## Author Summary

After a meal, dietary glucose travels through the hepatic portal vein to the liver. A substantial part of this glucose is taken up by liver, which converts it to glycogen which is stored, and lipids which are in part stored and in part secreted as VLDL particles. The rest of the organs receive whatever glucose the liver leaves in circulation, plus the secreted lipids. Hence the liver plays a crucial role in determining the balance of sugar versus lipids in the body after a meal. This balance is very important, because too much glucose in circulation leads to diabetic complications whereas too much VLDL increases risk of atherosclerosis. Little is known about how the liver strikes this balance. We identify here a phosphatase—the PP2A holoenzyme containing the PPP2R5C regulatory subunit—as a regulator of this process. We find that knockdown of PPP2R5C in mouse liver specifically causes it to uptake elevated levels of glucose, and secrete elevated levels of VLDL into circulation. This leads to a phenotype of improved glucose tolerance and insulin sensitivity. The prediction from these functional studies in mice is that elevated levels of PPP2R5C expression should lead to insulin resistance. Indeed, we find that PPP2R5C expression levels are elevated in diabetic patients, or healthy controls with visceral obesity, raising the possibility that dysregulation of PPP2R5C expression in humans may contribute towards metabolic dysfunction.

## Introduction

According to Greek mythology, Odysseus was forced to carefully navigate his ship between two dangers—Scylla and Charybdis—whereby passing too close to either one would lead to destruction. Likewise, in metabolic regulation, the liver strikes a difficult balance between glucose and lipid homeostasis. After a meal, dietary glucose travels through the hepatic portal vein to the liver. The liver uptakes a substantial part of this glucose, removing it from circulation, and converting it to glycogen for storage, or to lipids which are in part stored and in part re-secreted as VLDL particles [1–3]. Hence the liver plays a crucial role in determining the balance of sugar versus lipids in the body after a meal. While elevated circulating glucose is intimately linked to diabetes, elevated lipids are linked to atherosclerosis or non-alcoholic fatty liver disease [4–6]. Despite the critical importance of handling both glucose and lipid pathways in a coordinated and balanced manner, the molecular mechanisms regulating this balance remain to be investigated. We describe here a phosphatase that affects how the liver strikes this balance between glucose and lipid metabolism.

Phosphatases are an interesting class of enzymes because they can have specific, yet very strong cellular effects by dephosphorylating multiple proteins in one signaling pathway or biological process [7–13]. One such phosphatase is the PP2A holoenzyme, the most abundant serine/threonine phosphatase in the cell. The PP2A holoenzyme is composed of three subunits, a large scaffolding A subunit, a catalytic C subunit which performs the dephosphorylation reaction, and one of many possible regulatory B subunits which provide substrate specificity to the holoenzyme [10,14]. Four families of B subunits have been identified (B, B’, B” and B”‘) with each family containing multiple members [10], resulting in a large array of possible B subunits. Hence, although the PP2A catalytic subunit which is shared by all these holoenzymes has pleiotropic effects, the individual B subunits which direct the phosphatase to a subset of targets can have very specific effects [15,16]. We previously showed that Drosophila lacking one of the B’ subunits, PP2A-B’, are viable but have metabolic defects [17]. Starting from these observations in the fly, we hypothesized that one of the two mammalian homologs of PP2A-B’, called PPP2R5C or B56**γ**, might also be involved in metabolic regulation in mammals.

To date, PPP2R5C has been linked to cancer development. Several studies have reported either increased or decreased expression of PPP2R5C in various tumor types [18–21]. One mechanism by which PPP2R5C affects tumor development appears to be via dephosphorylation of p53 on Thr55 [22–24], leading to inhibition of cell proliferation and anchorage-independent growth [23,25]. PPP2R5C has also been proposed to have p53-independent mechanisms [26]. Indeed, mouse p53 appears to lack Thr55, suggesting that PPP2R5C acts via additional mechanisms. Whole-body PPP2R5C knockout mice are viable but display heart defects including an incomplete ventricular septum and reduced numbers of ventricular cardiomyocytes [27]. PPP2R5C knockout mice also display reduced locomotive coordination and gripping strength [27]. Together with the heart defects, this suggests a muscular function for PPP2R5C. Interestingly, PPP2R5C knockout mice also developed obesity with age [27], raising the possibility that PPP2R5C regulates metabolism either directly or indirectly as a consequence of its effects on locomotion. The involvement of PPP2R5C in the regulation of metabolic homeostasis, however, remains to be investigated.

We investigate here for the first time the consequences of tissue-specific PPP2R5C deficiency, and show that hepatic PPP2R5C plays a role in postprandial physiology, affecting the balance of conversion of sugar into lipids by the liver. PPP2R5C knockdown in hepatocytes (HepKD) leads to increased glucose uptake and lipid biosynthesis. Consequently, HepKD mice are highly glucose tolerant and insulin sensitive. Interestingly, we find that liver and adipose PPP2R5C expression levels correlate with diabetic state and glucose tolerance in humans in agreement with the functional studies in mouse models.

## Results

### Expression of PPP2R5C is regulated in response to nutritional and metabolic status

**Fig 1:**
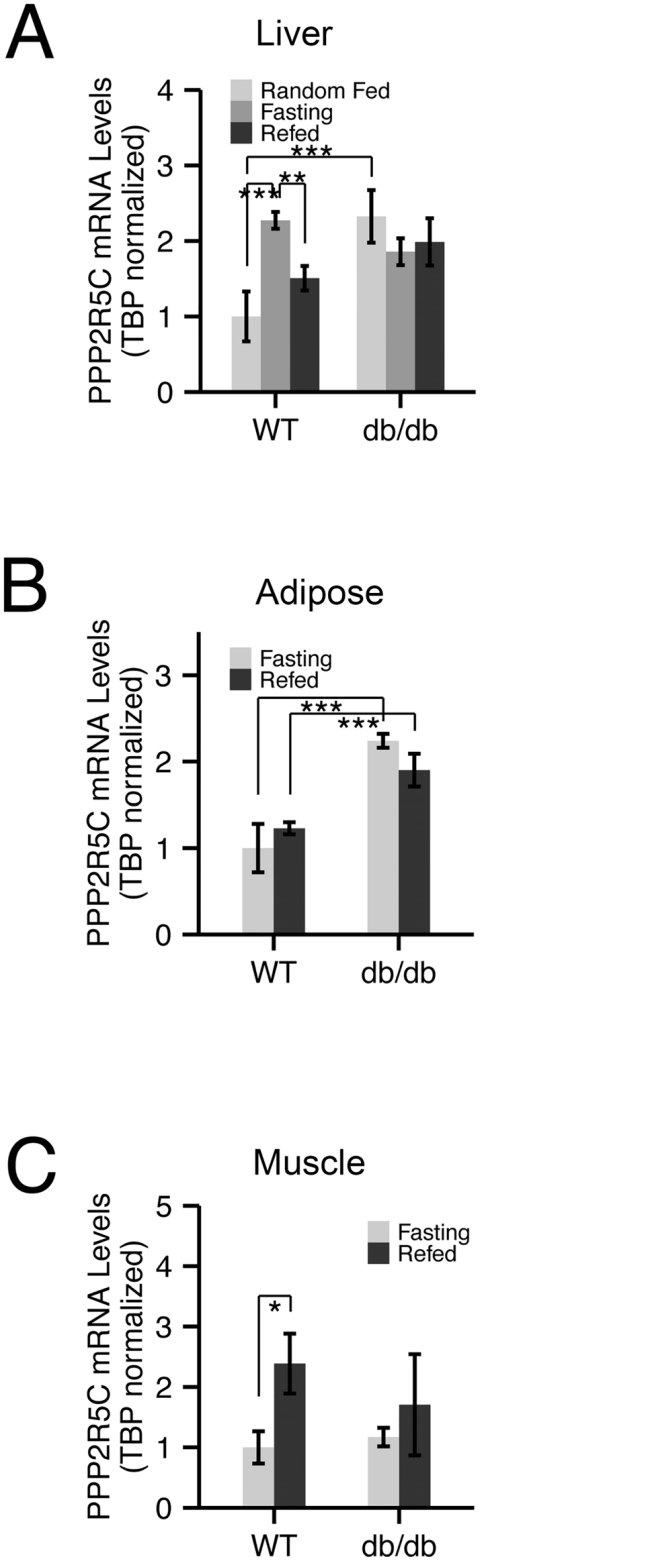
Expression of PPP2R5C is nutritionally regulated in metabolically relevant tissues. (A-C) mRNA levels of PPP2R5C are nutritionally regulated in various mouse tissues. Quantitative RT-PCR of PPP2R5C (NCBI splice variant 4) from liver (A), abdominal white adipose tissue (B) and gastrocnemius muscle (C) of control C57BL/6 (“WT”) or diabetic leptin-receptor deficient (“db/db”) 8–10 week old male mice. Mice were first starved for 16 hours (“Fasting”) and then given normal chow diet for 6 hours (“Refed”). Error bars: std. dev. *p-value<0.05, **p-value<0.01, ***p-value<0.001 by student t-test (n ≥ 3).

Since our previous studies in Drosophila identified a role for fly PPP2R5C (called PP2A-B’) in the regulation of organismal metabolism [17], we asked if we could also observe a link between PPP2R5C and metabolism in mice. Genes for metabolic regulators are often present in transcriptional regulatory feedback loops [28,29], so we first tested if organismal nutritional status affects PPP2R5C expression. Indeed, we found that PPP2R5C expression is nutritionally regulated in tissues of metabolic relevance such as liver, adipose tissue, and muscle, albeit in a complex way. In mouse liver, PPP2R5C expression increases upon fasting, and drops again upon refeeding (Fig 1A). This nutritional regulation of PPP2R5C is lost in obese diabetic db/db mice, which express elevated, constant levels of PPP2R5C. In abdominal white adipose tissue, PPP2R5C expression does not respond to changes in feeding status, but similar to liver, expression of PPP2R5C is significantly, tonically elevated in db/db mice compared to controls (Fig 1B). Opposite to liver, in gastrocnemius muscle PPP2R5C expression increased upon feeding (Fig 1C) and again this regulation is blunted in db/db mice (Fig 1C). These transcriptional responses to nutritional status did not translate into detectable changes in PPP2R5C protein levels in mouse liver, indicating that they are either buffered at the translational level, or our antibody is not sensitive enough to detect changes of this magnitude (S1A Fig). By screening a panel of drugs, we found that PPP2R5C expression is inhibited by stimulation with insulin or human FGF19 (homologous to mouse FGF15) in primary hepatocytes (S1B Fig) and is induced by a PPAR**α** agonist to a degree similar to that of a canonical PPARa target gene, CPT1A (S1C Fig). In contrast, stimulation of primary hepatocytes with leptin did not cause a change in PPP2R5C expression (S1D Fig) whereas feeding mice a high-fat diet for 4 weeks led to a mild increase in PPP2R5C expression (S1E Fig), suggesting that the elevated expression of PPP2R5C in db/db mice might be an indirect consequence of their altered metabolic status, and not directly due to impaired leptin signaling. In sum, although these transcriptional changes may not result in functional consequences, they suggest there might be links between PPP2R5C and tissue-specific metabolic regulation.

### Liver knockdown of PPP2R5C leads to increased glucose uptake and improved glucose tolerance

**Fig 2:**
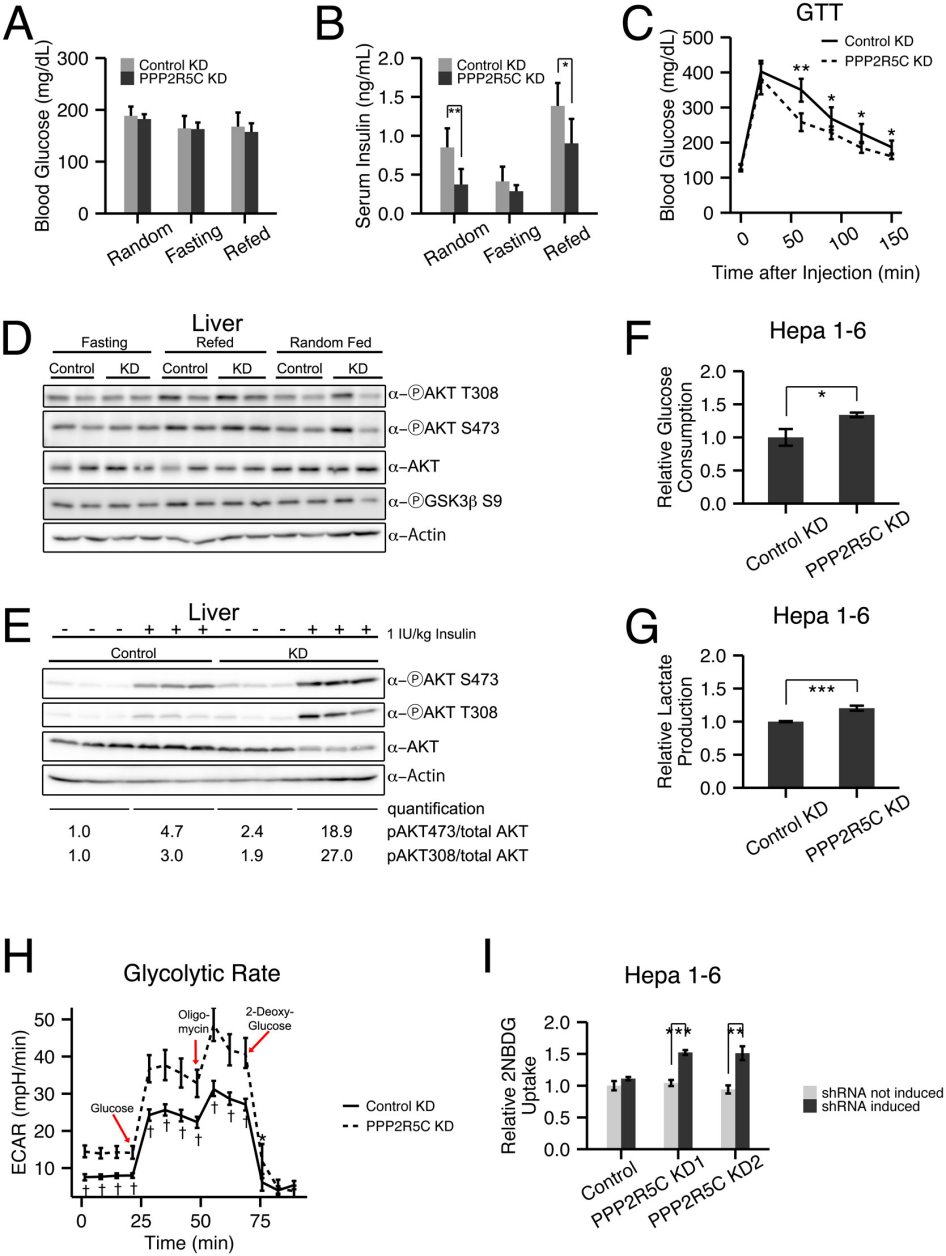
Hepatocyte-specific knockdown of PPP2R5C leads to increased glucose uptake and improved glucose tolerance. **(A-C)** Increased glucose uptake/tolerance in mice upon liver-specific knockdown of PPP2R5C. 8–10 week CL57BL/6 male mice tail-injected with adeno-associated virus containing miRNA targeting PPP2R5C (“PPP2R5C KD”) or a scrambled control miRNA (“Control KD”). 7 weeks after knockdown mice were starved for 16 hours (“Fasting”) or starved and then given normal chow diet for 6 hours (“Refed”) prior to sacrificing. Although blood glucose levels are not altered (A), serum insulin levels are significantly reduced (B) compared to controls. (C) Glucose tolerance test performed 4 weeks after virus injection shows significantly improved tolerance in knockdown mice (2g glucose/kg body weight injected intraperitoneally, n = 12). **(D)** Insulin signaling, detected via AKT and GSK3 beta phosphorylation, does not drop in all feeding regimes in PPP2R5C HepKD livers compared to controls, despite PPP2R5C HepKD serum insulin levels being lower (see panel B). **(E)** Insulin sensitivity is increased after PPP2R5C knockdown in liver. Control C57BL/6 mice and PPP2R5C HepKD mice were virus-injected as in Fig 2. Four weeks later, mice were fasted for 6 hours, then insulin was injected 1IU/kg and 10 minutes later mice were sacrified and liver samples were taken. **(F-G)** Glucose consumption and lactate production are increased in Hepa 1–6 cells upon PPP2R5C knockdown. Hepa 1–6 cells infected by adenovirus carrying shRNA targeting all mouse PPP2R5C isoforms (PPP2R5C KD) or a negative-control scramble shRNA (Control KD). After 48h, glucose consumption (F) and lactate production (G) were measured in the medium for 24 hours, and normalized to total cell protein. (n = 3) **(H)** Glycolytic flux measured as ECAR (extracellular acidification rate) using the glycolysis stress kit from Seahorse Bioscience on the extracellular flux analyser XF96. After addition of glucose to control or PPP2R5C knockdown Hepa 1–6 cells, oligomycin is added to inhibit respiration, thereby boosting glycolytic flux. 2-deoxy-glucose is added to compete with glucose and shut down glycolysis (n = 9). **(I)** Acute glucose uptake is increased in Hepa 1–6 cells upon PPP2R5C knockdown. Stably transfected Hepa1-6 cell-lines carrying two independent, inducible shRNAs (PPP2R5C KD1 and KD2) were induced with 30 μg/ml cumate for 3 days, starved overnight in serum-free DMEM, and uptake of fluorescent 2-deoxy-glucose analog 2-NBDG was quantified by FACS. (n = 3) Error bars: std. dev. *p-value<0.05, **p-value<0.01, ***p-value<0.001, †p-value<10^−4^ by Wilcoxon signed-rank test (C) or student t-test (B, F-I).

To study if PPP2R5C regulates metabolism in mammals, we knocked-down PPP2R5C expression in vivo in the mouse and assayed if this leads to metabolic alterations. Since liver is a central organ for metabolic regulation, we focused specifically on hepatic PPP2R5C function. Tail-vein injection of adeno-associated virus carrying a miRNA under control of a hepatocyte-specific promoter [30] targeting all transcriptional isoforms of PPP2R5C led to a significant reduction in PPP2R5C mRNA and protein levels in the liver (S2A–S2A’ Fig). As a control, we injected equal amounts of an adeno-associated virus carrying a non-targeting miRNA. Body weight and serum ALT levels were not altered upon PPP2R5C hepatocyte-specific knockdown (“HepKD”) (S2B and S2C Fig), indicating that the liver is not experiencing severe stress in these conditions. Since the liver is important for maintaining euglycemia, we measured blood glucose levels in knockdown mice either in an uncontrolled feeding regimen (“Random”), or after 16 hours of fasting (“Fasting”), followed by 6 hours of refeeding (“Refed”). In all three conditions, PPP2R5C HepKD did not have a significant effect on blood glucose levels (Fig 2A). These same mice, however, displayed dramatically reduced levels of circulating insulin (Fig 2B), indicating that lower insulin levels are required to maintain euglycemia upon liver knock-down of PPP2R5C. Consistent with this, PPP2R5C HepKD mice defended circulating glucose levels more efficiently than control mice in a glucose tolerance test (2g glucose injected intraperitoneally per kg body weight, Fig 2C) despite lower insulin levels (S2D Fig). In sum, these data suggest PPP2R5C HepKD livers have elevated insulin sensitivity. Indeed HepKD livers maintain activation of insulin signaling, as judged by phosphorylation of Akt and the downstream target GSK3**β**, despite lower circulating insulin levels (Fig 2D and 2B). Strikingly, in a direct test of insulin sensitivity by measuring Akt phosphorylation 10 minutes after tail-injection of insulin we found that indeed PPP2R5C HepKD livers have elevated insulin sensitivity (Fig 2E).

To test if this is a cell-autonomous phenotype in the liver, we turned to cell culture. Knockdown of PPP2R5C (S2E Fig) caused Hepa 1–6 cells to deplete glucose from the medium more quickly than control knockdown cells (Fig 2F). Correspondingly, lactate production was also elevated in Hepa 1–6 cells upon PPP2R5C knockdown, indicating an increase in the overall glycolytic flux (Fig 2G). This was confirmed by measuring glycolytic flux using a Seahorse analyzer (Fig 2H). We also observed an increase in glucose uptake following a paradigm similar to the Glucose Tolerance Test, whereby Hepa 1–6 cells were starved overnight in serum-free DMEM and then treated with a fluorescent glucose analog for 20 minutes. Quantification by FACS revealed increased uptake upon induction of two independent shRNAs targeting PPP2R5C (Fig 2I, S2F Fig for knockdown efficiency control), thereby also ruling out possible off-target effects. Together, these data indicate that knockdown of PPP2R5C leads to a cell-autonomous increase in glucose uptake.

In addition to increased hepatic glucose uptake, one other factor that could contribute to improved glucose tolerance in vivo is reduced gluconeogenesis. However intraperitoneal injection of pyruvate, a gluconeogenic substrate, caused a similar ascension in blood glucose levels of PPP2R5C HepKD mice compared to controls (0–50 min, S2G Fig), indicating they do not have significantly reduced gluconeogenic capacity. Consistent with improved glucose clearance in PPP2R5C HepKD mice, blood glucose levels returned to baseline more quickly in knockdown mice compare to controls (the descending phase of the pyruvate tolerance test, S2G Fig), although the difference was not statistically significant. Consistent with the lack of change in gluconeogenic capacity, we also found no reduction in expression of gluconeogenic genes in PPP2R5C HepKD mice (S2H Fig). We did observe an increase in expression of G6PC (S2H Fig), which is a ChREBP target [31], as discussed below.

In sum, knockdown of PPP2R5C in liver leads to increased glucose uptake, improved glucose tolerance, improved insulin sensitivity and reduced insulin levels in vivo.

### PPP2R5C liver knockdown leads to increased TAG biosynthesis and VLDL secretion

**Fig 3:**
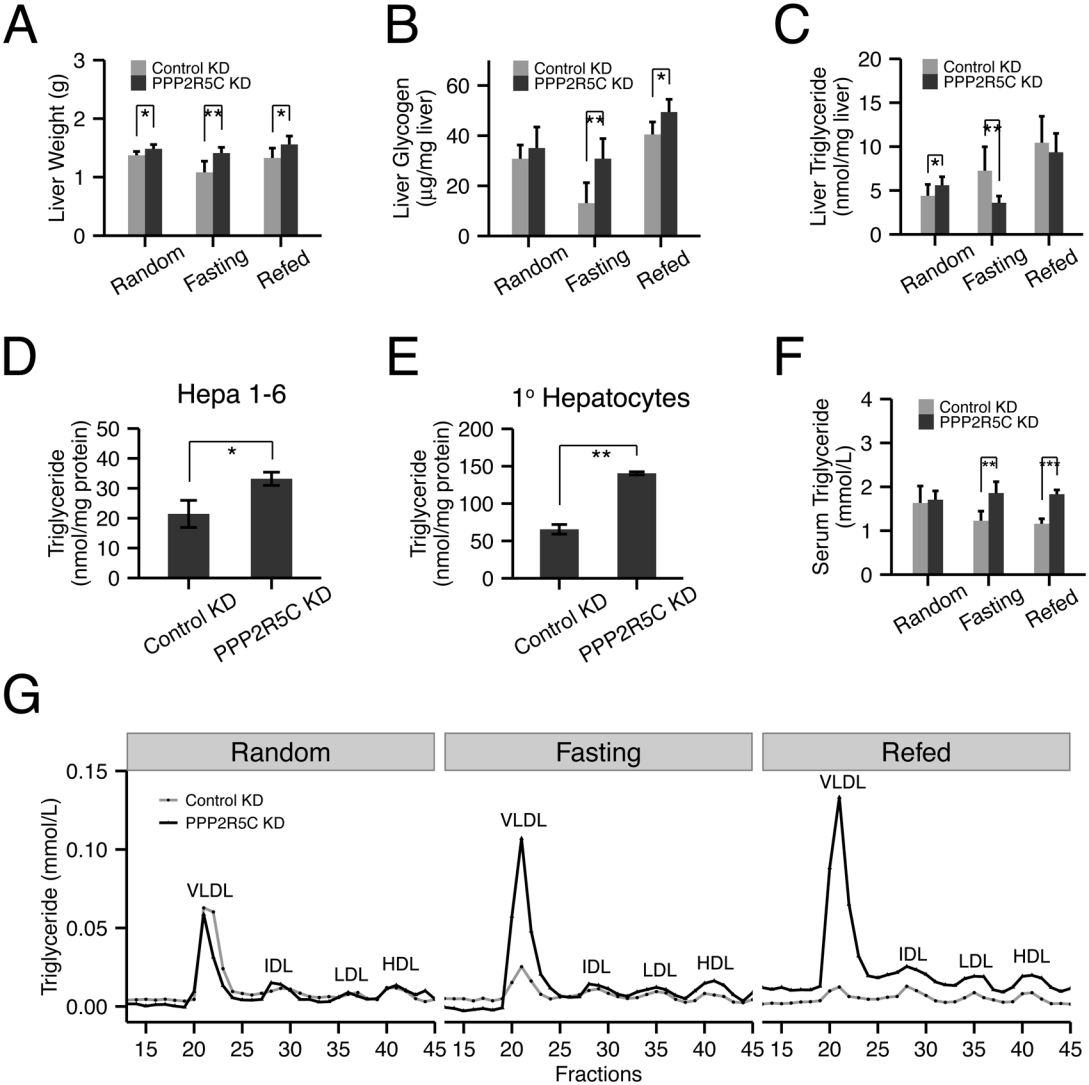
Hepatocyte-specific knockdown of PPP2R5C leads to increased lipogenesis and lipid secretion. **(A-C)** Liver-specific knockdown of PPP2R5C leads to pro-anabolic changes including increased liver lipid synthesis and secretion and reduced glycogen breakdown. As in Fig 2, 7 weeks post hepatocyte-specific knockdown, liver weight (A), glycogen (B) and triglycerides (C) were quantified. (n = 5 or 6) **(D-E)** Cellular triglyceride levels are increased in Hepa 1–6 (D) or mouse primary hepatocytes (E) upon PPP2R5C knockdown. Cells infected as in Fig 2. (n = 3) (**F-G**) Liver-specific knockdown of PPP2R5C leads to elevated serum VLDL levels. 7 weeks post hepatocyte-specific knockdown, serum triglycerides were quantified either in aggregate (F), or when fractionated by FPLC to resolve lipoprotein particles of various densities (G). (n = 5 or 6) Error bars: std. dev. *p-value<0.05, **p-value<0.01 by one-way ANCOVA with liver NEFA as a covariate (C, “Random”) or student t-test (A-F).

The increased glucose uptake upon PPP2R5C knockdown suggests that PPP2R5C knockdown might cause liver cells to shift towards a more anabolic metabolic profile. In agreement with this, quantification of liver weight revealed that PPP2R5C HepKD leads to increased liver mass in all three feeding regimens tested (Fig 3A), despite normal food intake (S3A Fig). Glucose is used by hepatocytes in part to synthesize glycogen [32]. Consistent with the increased glucose uptake, PPP2R5C knockdown livers had increased glycogen levels compared to controls, even when normalized to liver weight (Fig 3B). Most strikingly, although glycogen levels drop in control livers upon fasting, as they enter a catabolic state to provide the rest of the organism with glucose, PPP2R5C knockdown livers displayed almost no drop in glycogen upon fasting (Fig 3B).

Glucose is also used by hepatocytes for lipid biosynthesis. In the random feeding state, mice with PPP2R5C HepKD had significantly elevated TAG levels in their livers. Although this effect was visible 7 weeks after injection of PPP2R5C knockdown virus (Fig 3C), it was even more pronounced 2 weeks after virus injection (S3B Fig), perhaps due to compensatory regulatory mechanisms developing over time. One possible explanation for the increased liver TAG levels could be reduced liver fatty acid beta-oxidation, however circulating ketone bodies were not elevated in HepKD mice, suggesting this is not the case (S3C Fig). Furthermore, PPP2R5C knockdown in Hepa 1–6 cells did not lead to reduced levels of fatty acid beta-oxidation (S3 Fig panel D), which if anything were slightly elevated. Alternatively, HepKD livers might have elevated lipid biosynthesis rates. To test if PPP2R5C knockdown leads to increased lipogenesis in a cell-autonomous manner in hepatocytes, we turned once again to cell culture. Both Hepa 1–6 cells as well as primary mouse hepatocytes displayed increased TAG levels upon PPP2R5C knockdown (Fig 3D and 3E). These increased TAG levels could not be explained by an increase in free fatty acid uptake from the medium (S3E Fig), nor by reduced TAG secretion into the medium (since neither control nor PPP2R5C knockdown Hepa 1–6 cells secrete TAG into the medium, S3F Fig), indicating that PPP2R5C leads to both increased de novo lipogenesis and triglyceride formation in hepatocytes. PPP2R5C HepKD livers had reduced triglyceride levels upon fasting, when de novo lipogenesis is very low (Fig 3C), as discussed below. Upon refeeding, however, they re-accumulated triglycerides more rapidly than controls, reaching control levels within 6 hours of refeeding (Fig 3C), consistent with elevated de novo lipogenesis in PPP2R5C knockdown livers when dietary glucose is available. Taken together, PPP2R5C knockdown livers take up more glucose than control livers, thereby producing more triglycerides,

Surprisingly, triglyceride levels dropped significantly in PPP2R5C HepKD livers upon fasting when de novo lipogenesis in liver is very low (Fig 3C). This was accompanied by a strong elevation of circulating VLDL levels in PPP2R5C HepKD mice upon fasting or brief refeeding (Fig 3F, 3G and S3G Fig). One possible explanation consistent with reduced liver triglycerides and increased circulating VLDL is that HepKD livers secrete more VLDL than controls. Indeed consistent with this explanation, PPP2R5C HepKD led to a concomitant drop in liver cholesterol levels upon fasting (S3H Fig), without a significant change in circulating free fatty acid levels (S3I Fig). However, other explanations for this phenotype are also possible, such as reduced VLDL re-uptake by HepKD livers, as discussed below.

In sum, PPP2R5C knockdown leads to increased glucose uptake and increased de novo lipogenesis in cell culture and in vivo when dietary glucose is available, and elevated VLDL in circulation upon starvation.

### PPP2R5C-containing PP2A holoenzyme acts via multiple metabolic regulators

**Fig 4:**
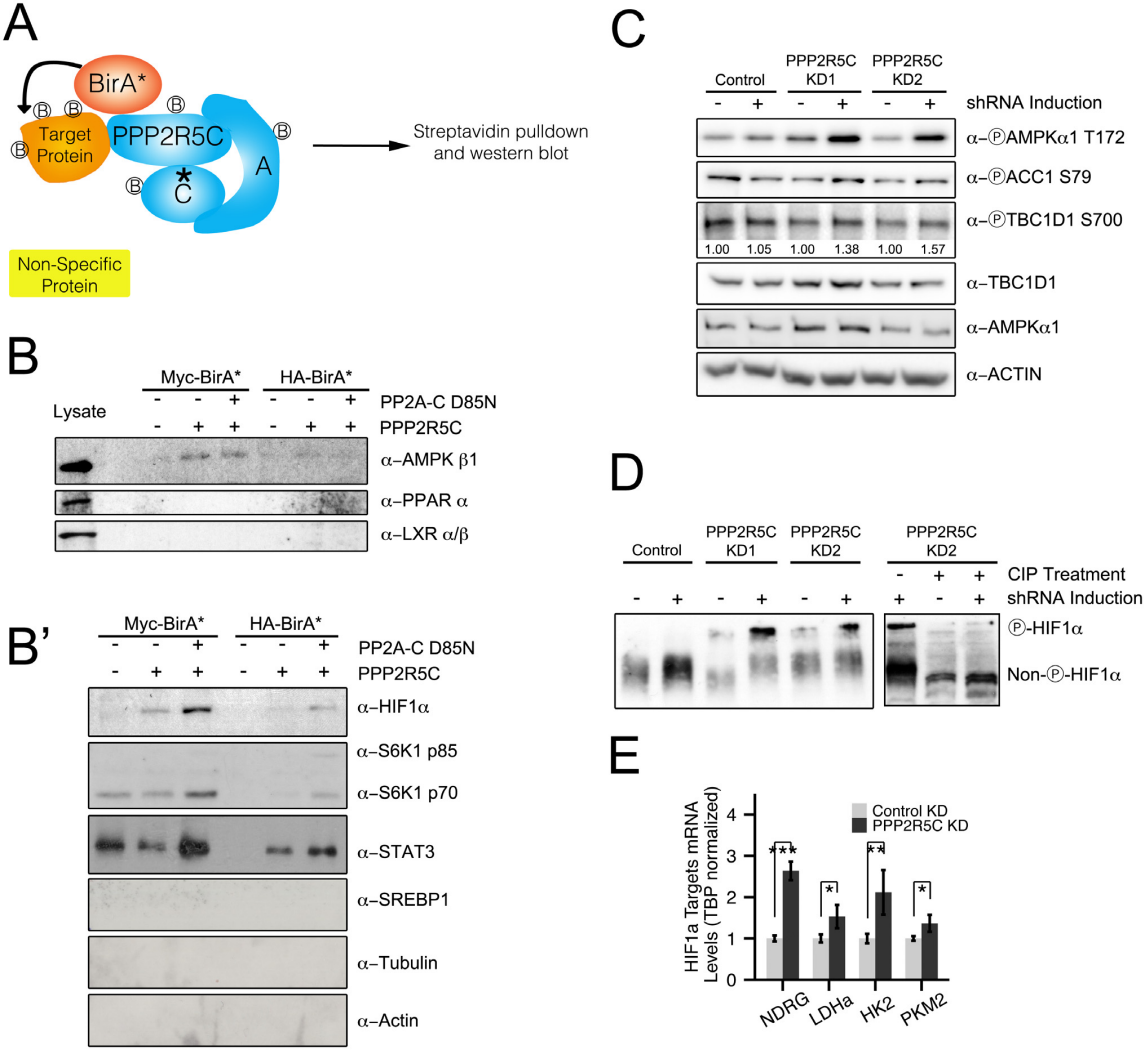
PPP2R5C regulates AMPK and HIF1α. **(A)** Schematic diagram of “BioID”—proximity-dependent biotinylation of proteins—to identify PPP2R5C substrates interacting transiently with the phosphatase complex. Biotin ligase (BirA) was fused to either the N-terminus (Myc-BirA-PPP2R5C) or C-terminus (PPP2R5C-BirA-HA) of PPP2R5C (NCBI variant 1). As negative controls, only BirA-HA or Myc-BirA were used. A mutation in the active center of the catalytic domain was introduced (PP2A-C D85N) as a substrate-trapping mutation. Biotinylated proteins from Hepa 1–6 cells were purified by cell lysis and streptavidin pulldown, and subsequently detected by immunoblotting. **(B-B’)** BioID identifies AMPK beta 1, HIF1**α**, STAT3 and S6K as PPP2R5C interacting proteins. Proteins interacting with PPP2R5C in vivo in Hepa 1–6 cells were biotinylated and purified as in (A), and probed by immunoblotting. LXR, PPAR alpha, SREBP-1, Actin, and Tubulin were included as negative control proteins which were not detected in the pulldowns. (Lysates shown at same exposure as biotin pulldowns.) **(C)** AMPK phosphorylation and activity increase upon PPP2R5C knockdown in Hepa 1–6 cells. PPP2R5C was knocked-down using two independent, inducible shRNAs as in Fig 2G. AMPK phosphorylation increases on T172 upon PPP2R5C knockdown. AMPK activity determined via phosphorylation of two substrates, ACC1 and TBC1D1. **(D)** HIF1α phosphorylation increases upon PPP2R5C knockdown in Hepa 1–6 cells. HIF1α phosphorylation is detected as reduced mobility (up) on a 25 μM PhosTag gel (8% gel), which is abolished upon treatment of cell lysates with calf intestinal phosphatase (CIP) prior to gel electrophoresis (right panel). PPP2R5C knockdown was performed by generating stable cell lines carrying two independent inducible shRNA constructs (KD1 and KD2) to exclude off-target effects. **(E)** Expression of HIF1**α** target genes increases in primary hepatocytes upon PPP2R5C knockdown. (n = 4) Error bars: std. dev. *p-value<0.05, **p-value<0.01, ***p-value<0.001 by student t-test (E).

PPP2R5C is a regulatory subunit of PP2A, thought to provide substrate specificity to the phosphatase holoenzyme. Therefore, we aimed to identify target substrates that bind PPP2R5C. PPP2R5C has been shown to target Thr55 of human p53 for dephosphorylation, but this residue is not present in mouse p53, prompting us to search for additional substrates. Since protein-protein interactions between phosphatases and substrates are notoriously transient and difficult to detect via co-immunoprecipitation strategies, we employed the BioID method [33] to identify PPP2R5C interacting proteins. We expressed in Hepa 1–6 cells a fusion between PPP2R5C and the biotin ligase BirA (Fig 4A). This leads to biotinylation in vivo of PPP2R5C interacting proteins, which can subsequently be purified by cell lysis and streptavidin binding. We fused BirA to either the N-terminus or the C-terminus of PPP2R5C (Myc-BirA-PPP2R5C and PPP2R5C-BirA-HA respectively), and used Myc-BirA or BirA-HA alone as negative controls. In addition, we also introduced a mutation into the catalytic subunit of PP2A (PPP2CA) known to eliminate phosphatase activity [34], with the aim of generating a ‘substrate-trapping’ mutation to extend the duration of interaction between PP2A and substrate proteins. In this manner, we tested if PPP2R5C binds to various known regulators of liver metabolism, and found specific interactions with the beta-1 subunit of AMPK (Fig 4B and S4A Fig), HIF1α, STAT3 and S6K (Fig 4B’). In contrast, we could detect no binding of PPP2R5C to SREBP-1, PPARα, LXR, or a panel of negative control proteins (RpL26, TSC1, YAP, HSP90, Tubulin, and Actin, Fig 4B–4B’, S4A Fig). Since phosphatases are known to dephosphorylate multiple substrates, these results suggest that the metabolic effects of PPP2R5C might be due to the combined effects on several metabolic regulators. We tried to confirm the interaction between PPP2R5C and AMPK by co-immunoprecipitation of endogenous proteins, but were unsuccessful, possibly due to the transient nature of phosphatase–substrate interactions which rarely survive biochemical purification. Instead, we employed Proximity Ligation Assay (PLA) [35] which detects protein-protein interactions in situ by fixing cells, staining with two antibodies recognizing the two proteins of interest, and detecting complex formation of the two antibodies. We could detect a strong PLA signal when tagged PPP2R5C and AMPK-**β**1 were expressed in Hepa 6–1 cells, but not when only one of the two proteins was expressed, indicating specificity of the interaction (S4B Fig).

We next tested if binding of PPP2R5C to AMPK or HIF1a leads to changes in their phosphorylation state and activity. If the PPP2R5C-PP2A holoenzyme dephosphorylates AMPK, we would expect increased AMPK phosphorylation upon PPP2R5C knockdown. To test this, we generated Hepa 1–6 cell lines transfected with two independent, inducible shRNAs targeting PPP2R5C. We used two independent shRNAs targeting PPP2R5C to avoid possible off-target effects. Upon induction of the PPP2R5C-targeting shRNAs, AMPK phosphorylation on Thr172 increased significantly (Fig 4C). Concurrently, phosphorylation of two AMPK substrates, ACC1 and TBC1D1, was also elevated upon PPP2R5C knockdown (Fig 4C) suggesting that PPP2R5C knockdown leads to elevated AMPK activity.

Since PPP2R5C also interacts with HIF1**α** (Fig 4B’), we next studied the effect of PPP2R5C knockdown on HIF1**α** phosphorylation. To our knowledge, however, phospho-specific antibodies are not available to detect HIF1**α** phosphorylation. Therefore, we employed phos-tag gels, which contain functional groups that specifically bind phosphate, causing phosphorylated proteins to migrate more slowly compared to their respective un-phosphorylated forms [36]. In this manner, we observed an up-shift of HIF1**α** upon PPP2R5C knockdown in Hepa 1–6 cells, that could be reversed by treating the cell lysates with CIP phosphatase prior to PAGE (Fig 4D), suggesting that PPP2R5C affects HIF1**α** phosphorylation. To test if this has any functional consequences, we looked at expression of HIF1**α** target genes and found them to be up-regulated in primary hepatocytes upon PPP2R5C knockdown (Fig 4E).

### PPP2R5C knockdown livers have elevated levels of SREBP-1, SREBP-1 target genes and ChREBP target genes

**Fig 5:**
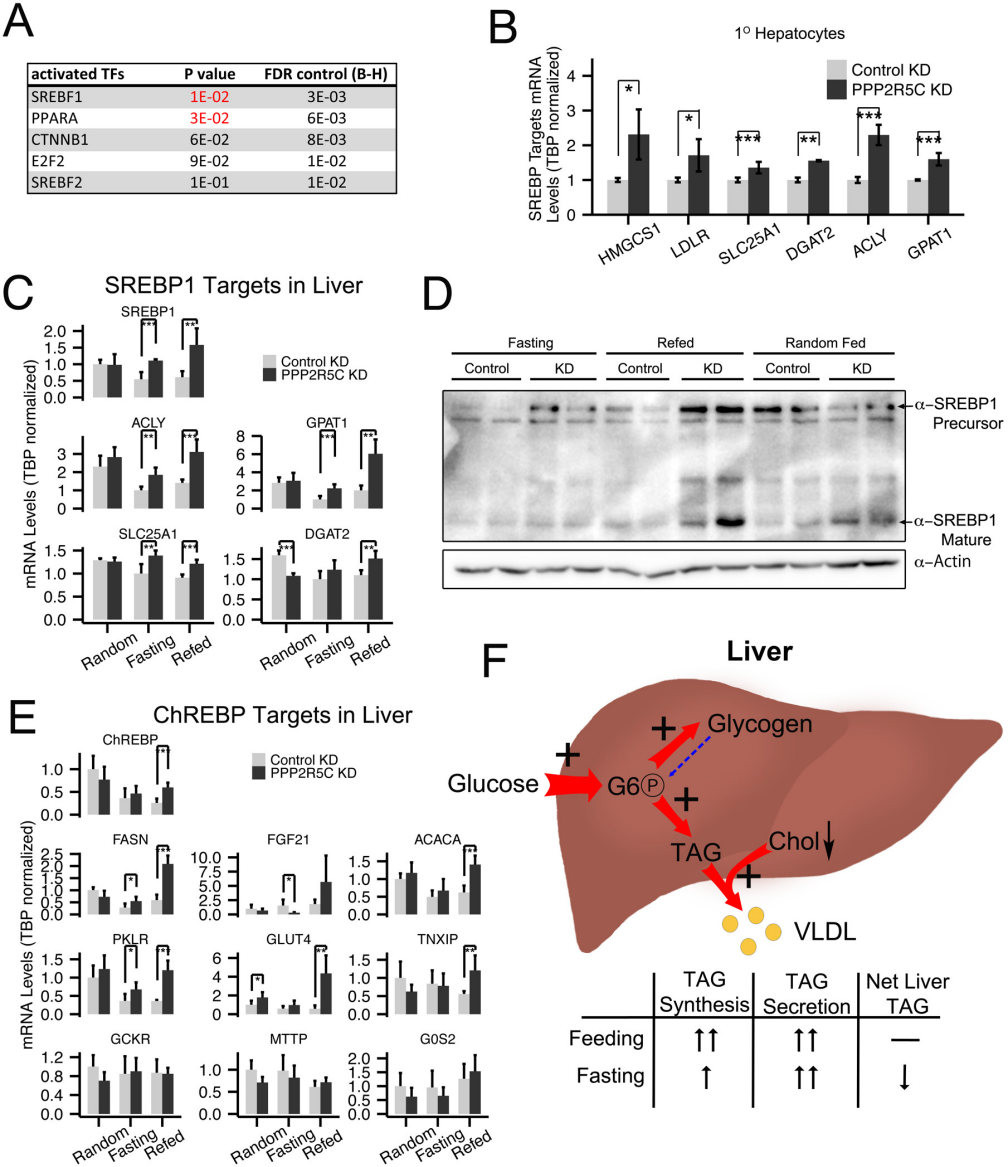
PPP2R5C knockdown leads to SREBP-1 and ChREBP activation. **(A)** PPP2R5C knockdown leads to upregulation of genes enriched for PPARA and SREBP-1 targets. Genes either up- or down-regulated upon PPP2R5C knockdown in mouse primary hepatocytes were analyzed using TFactS software [37] to identify transcription factors putatively misregulated upon PPP2R5C knockdown. FDR (False Discovery Rate) rate was controlled using the Benjamini-Hochberg procedure. **(B-C)** Expression of bona-fide SREBP-1 target genes is increased upon PPP2R5C knockdown in primary hepatocytes in culture (B) or in mouse liver in vivo (C). PPP2R5C was knocked-down in mouse primary hepatocytes using adenovirus and in vivo using adeno-associated virus as in Fig 2. SREBP-1 target genes quantified by Q-RT-PCR, normalized to TBP. **(D)** Upon PPP2R5C knockdown in liver, SREBP-1 protein levels are elevated. **(E)** Expression of ChREBP target genes is increased upon PPP2R5C knockdown in mouse liver in vivo. PPP2R5C was knocked-down in vivo using adeno-associated virus as in Fig 2. ChREBP target genes quantified by Q-RT-PCR, normalized to TBP. **(F)** Graphical representation of the metabolic changes induced upon PPP2R5C knockdown in mouse liver. Livers with reduced PPP2R5C have increased glucose uptake, increased TAG synthesis, and increased VLDL secretion. Error bars: std. dev. *p-value<0.05, **p-value<0.01, ***p-value<0.001 by student t-test (B-C,E) (n = 4 for mouse primary hepatocytes, and 5 or 6 for mouse liver).

To analyze more broadly the changes occurring within hepatocytes upon PPP2R5C knockdown, we performed microarray expression profiling of polyA mRNA from mouse primary hepatocytes in the presence and absence of PPP2R5C knockdown. This identified 11 down-regulated and 49 up-regulated genes upon PPP2R5C knockdown (2-fold cut-off, S1 Table). Since PPP2R5C is part of a phosphatase complex, these transcriptional changes likely occur as a secondary consequence of altered activity of transcription factors in signaling pathways targeted by PPP2R5C. To identify these transcription factors, we used the TFactS software, which predicts transcription factors that are dysregulated by comparing lists of up-regulated and down-regulated genes to annotated catalogs of transcription factor target genes [37]. This analysis identified PPAR**α** and SREBP-1 as the two up-regulated transcription factors upon PPP2R5C knockdown (p-value<0.05, Fig 5A). Since SREBP-1 promotes lipid biogenesis, which is up-regulated in PPP2R5C knockdown hepatocytes (Fig 3), we analyzed this in more detail. Knockdown of PPP2R5C in primary hepatocytes led to elevated expression of a panel of SREBP-1 target genes (Fig 5B). Elevated expression of SREBP-1 target genes was also observed in vivo in PPP2R5C knockdown livers, especially in the refed condition (Fig 5C), suggesting elevated SREBP-1 activity. PPP2R5C knockdown livers also had elevated levels of SREBP-1 precursor as well as mature SREBP-1 protein (Fig 5D), in agreement with SREBP-1 positively auto-regulating its own expression [29]. In sum, elevated SREBP-1 activity may be contributing to the increased lipogenesis phenotype of PPP2R5C knockdown hepatocytes.

Another transcription factor promoting conversion of carbohydrates into triglycerides in liver is ChREBP [38]. We found that PPP2R5C HepKD livers have elevated expression of several but not all ChREBP targets (Fig 5E and G6PC in S2H Fig), suggesting ChREBP activity might also be elevated in knockdown livers. In contrast, we did not see strong changes in expression of PPAR**α** target genes in knockdown livers compared to controls (S5 Fig panel A), although a few PPAR**α** target genes such as CPT1A and ADFP were significantly reduced. Finally, expression of two LXR targets was increased, but not expression of LXR itself (S5B Fig).

Taken together, these data suggest that PPP2R5C regulates AMPK, HIF-1**α**, and a yet-to-be identified target that affects SREBP-1 activity.

### Liver PPP2R5C expression correlates with obesity and insulin resistance in patients

**Fig 6:**
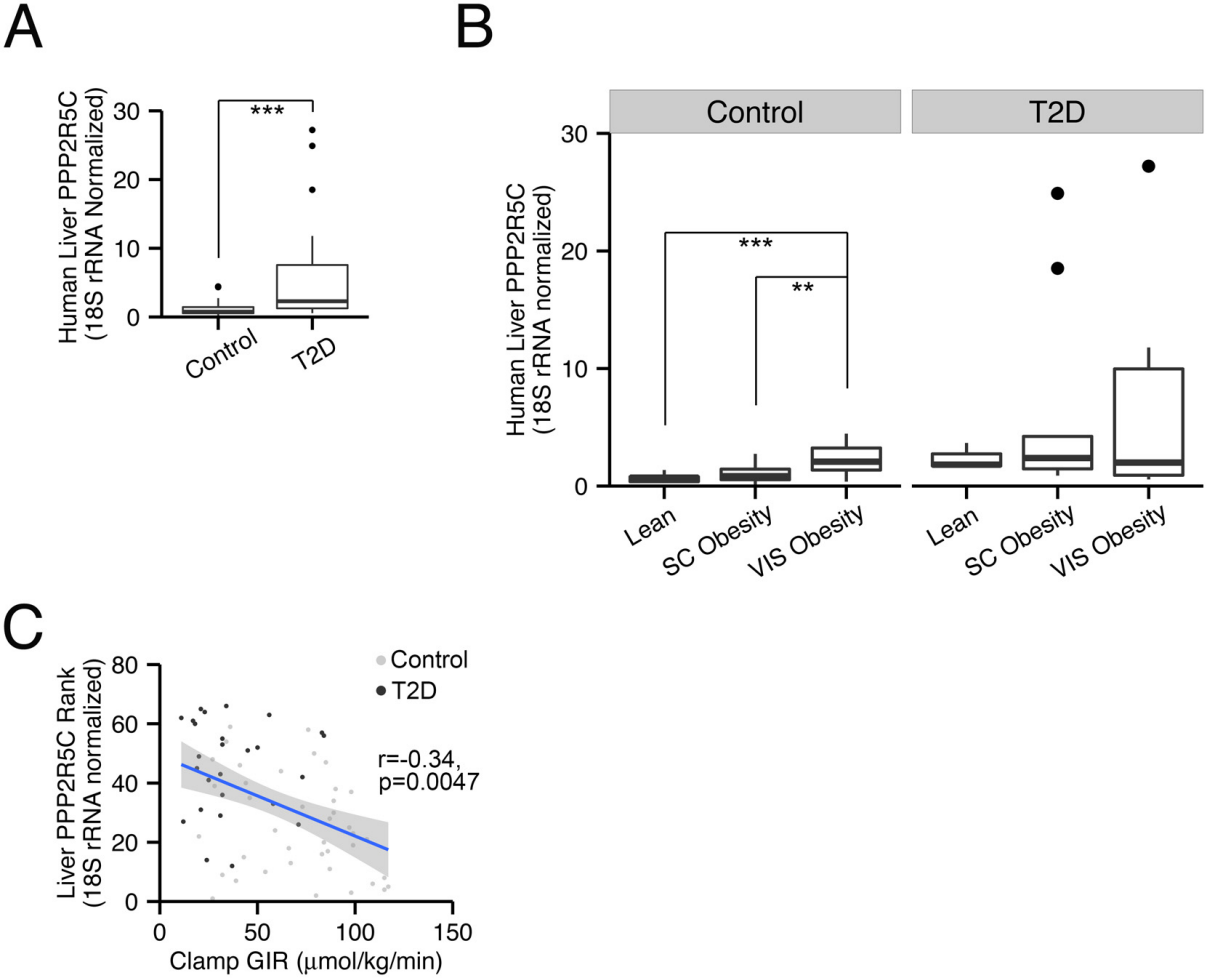
PPP2R5C expression in human liver correlates with insulin resistance. **(A)** Expression of PPP2R5C is elevated in livers of diabetic patients. Quantitative RT-PCR of Human PPP2R5C in liver of healthy controls (n = 40) or type 2 diabetic patients (n = 26), normalized to 18S rRNA. **(B)** Expression of PPP2R5C is elevated in non-diabetics with visceral obesity. People enrolled in the analysis were divided into 3 subgroups according to their adiposity: Lean (n = 12), Subcutaneous (“SC”) Obesity (n = 21) and Visceral (“VIS”) Obesity (n = 7). **(C)** Expression of PPP2R5C inversely correlates with insulin sensitivity. Pearson correlation analysis was done for PPP2R5C expression levels and glucose infusion rate (GIR) during hyperinsulemic-euglycemic clamp. **p-value<0.01, ***p-value<0.001 by student t-test (A-B).

Since reduced PPP2R5C expression in liver leads to significantly improved glucose tolerance and improved insulin signaling (Fig 2C and 2E), we wondered if diabetic patients might have the opposite—elevated levels of PPP2R5C expression. Indeed, PPP2R5C expression was significantly increased in livers of type-2 diabetic patients (p = 0.0003, Fig 6A). Even in non-diabetic patients, PPP2R5C expression increased with increasing adiposity, in particular visceral obesity, an important risk factor for diabetes (Fig 6B). Both in diabetic and non-diabetic patients, PPP2R5C liver expression correlated inversely with insulin sensitivity, determined by glucose infusion rate (GIR) during hyperinsulemic-euglycemic clamp (Fig 6C), in agreement with our mouse data showing that reduced PPP2R5C liver expression leads to improved glucose tolerance (Fig 2C). In sum, these data raise the possibility that altered PPP2R5C expression in liver might contribute towards the etiology of type-2 diabetes. We extended our analysis to other tissues and found that PPP2R5C expression is also elevated in subcutaneous, but not visceral, white adipose tissue in type-2 diabetic patients compared to healthy controls (p = 0.04, S6 Fig). These data fit nicely with the expression data from mice, showing elevated levels of PPP2R5C expression in livers and adipose tissue of diabetic mice (Fig 1A and 1B).

### PPP2R5C HepKD improves glucose metabolism but worsens lipid metabolism in diabetic mice

**Fig 7:**
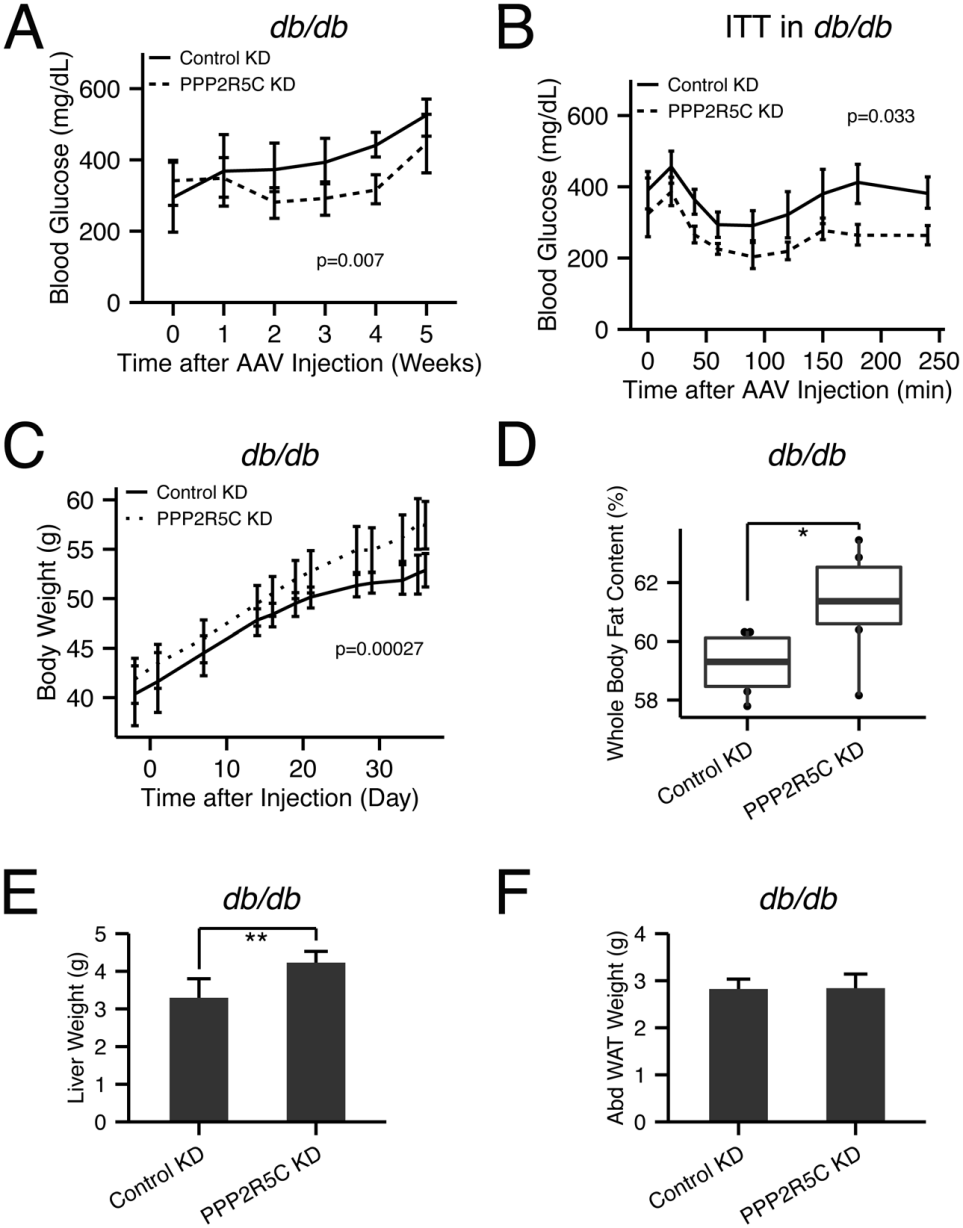
PPP2R5C HepKD in *db/db* mouse liver improves insulin sensitivity, decreases hyperglycemia, but worsens the dyslipidemia. **(A)** Hyperglycemia in *db/db* mice is decreased upon hypatocyte-specific PPP2R5C knockdown with adeno-associated virus. After 5 weeks knockdown, *db/db* mice were sacrificed under *ad libitum* feeding with normal chow diet. Blood glucose was monitored at each week, week 0 was one day before virus injection (n = 6). **(B)** Insulin tolerance test shows improved insulin sensitivity in PPP2R5C knockdown *db/db* mice at week 4 after virus injection (1.5IU/kg insulin was tail-injected after 6 hour fasting) (n = 6). **(C-F)** PPP2R5C HepKD in *db/db* mice increases body weight (C), whole body fat content (D), and liver weight (E), without changing abdominal adipose tissue weight (Abd.WAT) (F) (n = 6). Error bars: std. dev. *p-value<0.05 and **p-value<0.01 by student t-test (D-E). p-value in the (A-C) was calculated by two-way ANOVA.

Since PPP2R5C HepKD leads to improved insulin sensitivity and glucose tolerance, we asked if PPP2R5C HepKD could have beneficial effects in db/db mice, which are hyperphagic and consequently become obese and diabetic [39]. Consistent with the results of PPP2R5C knockdown in wildtype mice, PPP2R5C HepKD in leptin receptor-deficient db/db mice ameliorated their diabetic phenotypes, reducing circulating glucose levels (Fig 7A) and improving their response in an insulin tolerance test (Fig 7B). PPP2R5C HepKD in wildtype mice, however, indicated that the down-side of improved glucose handling is elevated liver or circulating triglycerides. Indeed, also in db/db mice, PPP2R5C HepKD led to elevated body mass accumulation (Fig 7C) and whole body fat content (Fig 7D) which was mainly due to increased triglycerides in liver (Fig 7E) but not adipose tissue (Fig 7F). In sum, PPP2R5C HepKD in diabetic mice worsened their dyslipidemia but ameliorated their hyperglycemia and improved their insulin response. This phenotype is interesting in light of the fact that obesity and insulin resistance, which often correlate in humans, can be uncoupled, with 20% of obese people displaying a ‘healthy obese’ phenotype with good insulin sensitivity and no diabetes [40].

## Discussion

We identify here PPP2R5C as a modulator of liver metabolism. Reduced PPP2R5C expression in hepatocytes leads to increased glucose uptake and increased de novo lipogenesis in cell culture. These phenotypes are reiterated in vivo whereby hepatocyte-specific knockdown (HepKD) of PPP2R5C yields mice with improved glucose tolerance but elevated liver triglyceride or circulating VLDL levels. Hence PPP2R5C modulates the balance the liver needs to strike between preventing circulating glucose levels from becoming too elevated after a meal, and yet not flooding the circulatory system with lipids (Fig 5F).

Interestingly, PPP2R5C HepKD mice have reduced levels of circulating insulin (Fig 2B) but normal levels of circulating glucose (Fig 2A). This is likely because the rheostat for euglycemia, the pancreas, is not affected in our HepKD mice. In the ‘random fed’ and ‘refed’ states, PPP2R5C HepKD livers uptake more glucose than normal (Fig 2). Upon starvation, PPP2R5C HepKD livers have reduced glycogen mobilization (Fig 3B) and normal gluconeogenesis (S2G Fig), indicating reduced glucose output in total compared to control animals. Hence, under all feeding conditions, PPP2R5C HepKD livers contribute towards reduced blood glucose (either via elevated glucose clearance or reduced glucose release) compared to control livers. Thus, to maintain euglycemia peripheral tissues such as muscle must be compensating by uptaking less glucose, likely as a result of reduced insulin from the pancreas.

The phenotypes we report here were obtained by targeting PPP2R5C with multiple different miRNA or shRNA sequences. For instance, the increased glucose clearance was observed in cell culture using two independent shRNAs (Fig 2G) and a third independent target sequence in vivo (Fig 2C). This allows us to exclude that the phenotypes could arise from possible off-target effects.

Although the phenotypes of PPP2R5C knockdown are quite specific both in cell culture and in vivo, they likely result from an effect of PPP2R5C on multiple downstream targets. Hence it will likely be difficult or impossible to identify a single downstream target as the main one mediating the effects of PPP2R5C. We identify here 4 protein complexes as PPP2R5C interactors—AMPK, HIF1a, STAT3 and S6K. We tested whether phosphorylation of these proteins increases upon PPP2R5C knockdown, as would be expected of a PPP2R5C target. For S6K, we analyzed phosphorylation on Thr389 using phospho-specific antibody, and did not find it elevated upon PPP2R5C knockdown in hepatocytes. For this reason, we did not pursue S6K further, although this does not exclude that S6K phosphorylation on another site or in a different cell type could be regulated by PPP2R5C. Indeed, the fact that PPP2R5C HepKD renders the liver more sensitive to insulin (Fig 2E) suggests it might be affecting insulin signaling by counteracting phosphorylation of a component of the insulin pathway. For STAT3, we analyzed phosphorylation of Ser727, but saw no change upon PPP2R5C knockdown. We also tested STAT3 motility on a phos-tag gel in an approach similar to what we did for HIF1**α** (Fig 4D), and did not observe any changes in its motility, although an important caveat from our experience is that phos-tag gels only resolve phosphorylations on roughly half of the proteins we have tested and know to be phosphorylated. For these reasons, we focused on AMPK and HIF1**α**. For AMPK and HIF1**α** we see both elevated phosphorylation and elevated activity upon PPP2R5C knockdown. Both AMPK and HIF1**α** can acutely increase glycolytic flux in response to stress conditions—either reduced energy supply or impaired mitochondrial function [41,42]–and therefore the two may act in concert to drive glucose uptake and glycolysis upon PPP2R5C knockdown. Indeed, the functional role of AMPK has been carefully studied in vivo in mouse liver, with increased liver AMPK activity leading to decreased blood glucose and fatty liver [43] and reduced liver AMPK activity leading to glucose intolerance and hyperglycemia during fasting [44], in agreement with what we see here. The phenotypes of liver-specific PPP2R5C knockdown and AMPK activation do not overlap completely, as expected if PPP2R5C also affects other metabolic pathways in addition to AMPK. It is unclear to what extent HIF1**α** could be contributing to the phenotypes observed in PPP2R5C HepKD mice, given that the mice we were studying were housed under normoxia. To our knowledge, whether HIF1**α** plays a role in mouse liver metabolism in such circumstances has not been studied. One study has shown that HIF1**α** can drive glycolysis and lipogenesis in cancer cells also under normoxic conditions [45]. Mice with liver-specific ablation of HIF1**β** develop diabetic phenotypes under normoxia [46], however HIF1**β** also binds other partners besides HIF1**α**. In our cell culture experiments with Hepa 1–6 cells, also conducted under normoxia, HIF1**α** is indeed functionally relevant because we can detect HIF1**α** protein by western blot (Fig 4B’ and 4D) and we see induction of HIF1**α** target genes upon PPP2R5C knockdown (Fig 4E). HIF1**α** is known to be frequently up-regulated and functionally relevant in cancers, therefore HIF1**α** may be a relevant downstream PPP2R5C target in this pathological context. Of note, however, although we see activation of AMPK and HIF1**α** upon PPP2R5C knockdown, due to technical limitations we have not been able to directly assay the contributions of these two targets to the PPP2R5C knockdown phenotype, for instance by performing genetic epistasis experiments. Further work will be required in this regard.

We observe activation of SREBP-1 in PPP2R5C HepKD livers, which likely contributes to their increased lipogenesis. SREBP-1 can be activated downstream of HIF1**α** [47] but additional mechanisms are likely to link PPP2R5C to SREBP-1 activation. We also observe increased expression of some, but not all ChREBP targets that we tested in PPP2R5C HepKD livers. This suggests elevated ChREBP activity could also be contributing to the increased lipogenesis in PPP2R5C HepKD livers. Unfortunately, however, we could not find a good antibody to detect endogenous ChREBP to test whether ChREBP might be a direct PPP2R5C target. Another important regulator of lipid metabolism is PPAR**α**, which ppromotes fatty acid beta-oxidation. Although we observed reduced expression of a few PPAR**α** targets, we do not see reduced fatty acid beta-oxidation in Hepa 1–6 cells upon PPP2R5C knockdown, suggesting that reduced PPAR**α** activity might not contribute to the PPP2R5C knockdown phenotype.

PPP2R5C has been linked to cancer development. One mechanism appears to be via dephosphorylation of p53 on Thr55 [22–24]. The results described here suggest that upon PPP2R5C knockdown, cells also increase their glucose uptake, glycolytic rate, and lipid biosynthesis—all of which are metabolic hallmarks for cancer cells. Therefore, it will be interesting to study in the future whether these metabolic effects might be contributing towards the tumor suppressive properties of PPP2R5C.

One phenotype requiring further investigation is that liver triglycerides drop in PPP2R5C HepKD mice upon starvation compared to the random feeding regimen, instead of increasing as they do in control animals. Upon starvation, adipose tissue mobilizes lipid stores, releasing them as free fatty acids into circulation. These are taken up by the liver and converted to triglycerides, leading to liver steatosis upon starvation. Together with the stored de-novo synthesized triglycerides, they are packaged and secreted as VLDL particles. Some of these particles are re-uptaken by the liver, whereas others travel to other organs. One explanation of the observed phenotype is that HepKD livers secrete elevated levels of VLDL, thereby depleting TAGs stored in liver. A second possible explanation is that PPP2R5C HepKD livers do not efficiently re-uptake VLDL that is in circulation, leading to reduced liver triglycerides and elevated VLDL. A third possible explanation is that PPP2R5C HepKD livers do not uptake sufficient fatty acids from circulation upon starvation. However, this does not explain why VLDL levels would be elevated, and would predict free fatty acid levels in circulation should be elevated upon starvation, which we do not see (S3I Fig). A fourth possible explanation is that peripheral organs in PPP2R5C HepKD mice uptake less VLDL upon starvation. This, however, does not explain why liver triglyceride levels drop in PPP2R5C HepKD mice, and would require a secreted signaling molecule from the knockdown liver to peripheral tissues to alter their behavior. A fifth possible explanation is the HepKD livers have elevated fatty-acid beta-oxidation, thereby depleting TAG stores. However, this also would not explain the elevated VLDL levels. Hence, in sum, we think the most likely explanations are that PPP2R5C HepKD livers either secrete elevated levels of VLDL or re-uptake reduced levels of VLDL, but further work will be required to look at this aspect carefully. A second phenotype requiring further investigation is that glycogen levels in PPP2R5C HepKD livers do not drop upon starvation as in control animals. Since our analysis of PPP2R5C targets by BioID was done in the ‘fed’ state (ie cells growing with glucose and insulin), a similar analysis in the ‘starving’ state might shed more light on both of these starvation-associated phenotypes.

We previously identified the fly homolog of PPP2R5C, PP2A-B’, as a metabolic regulator in the fly, which dephosphorylates S6K [17]. Interestingly, there are both similarities and differences between our results in the fly and in the mouse. One similarity is that PPP2R5C and PP2A-B’ both appear to bind S6K. One difference is that we do not observe an increase in S6K phosphorylation upon PPP2R5C knockdown in mouse hepatocytes. Since we previously observed an increase in S6K phosphorylation upon PPP2R5C knockdown in HeLa cells [17], this difference is less likely due to evolutionary divergence and more likely due to incomplete PPP2R5C knockdown in our setup. A second similarity is that both in mice and in flies PPP2R5C/PP2A-B’ regulates organismal metabolism. However, PPP2R5C liver knockdown has opposite consequences to the full-body PP2A-B’ knockout which makes flies lean. This may be due to tissue-specific effects. For instance, PP2A-B’ knockdown in fly muscle might contribute towards fly leanness be elevating metabolic rate, as could be expected by elevated S6K activity. If this effect predominates in the whole fly knockout, it would make the fly lean. Further work will be required to further understand these similarities and differences.

Our knockdown experiments show that PPP2R5C expression in liver inhibits glucose uptake and reduces insulin sensitivity. Astoundingly, we find that increasing PPP2R5C expression in human liver also correlates with insulin resistance. Type-2 diabetic patients have significantly elevated PPP2R5C liver expression levels compared to controls (Fig 6A). In fact, even within the control population PPP2R5C expression correlates with reduced insulin sensitivity (Fig 6C), raising the hypothesis for future studies that increased PPP2R5C expression might play a causative role in insulin resistance.

In sum, we identify we identify here PPP2R5C as a novel metabolic regulator in the liver.

## Materials and Methods

### Virus production

For transient cell culture knockdowns, shRNA targeting a common region of all PPP2R5C transcript variants, or a non-targeting scrambled shRNA, were packaged into adenovirus by using the BLOCKiT adenoviral RNAi system from Invitrogen (K4941-00) and produced in HEK293A cells. Adenovirus was purified from HEK293A lysates with a CsCl gradient and checked for titer using a TCID50 assay. For hepatocyte-specific PPP2R5C knockdown in mouse liver, miRNA targeting PPP2R5C, or a non-targeting scrambled miRNA, were packaged into adeno-associated virus (AAV) as described [30,48,49]. AAV production and purification was done by Vector Biolabs. Target sequences are listed in a table below.

### Inducible shRNA stable cell lines

Two independent shRNAs were cloned into an inducible piggyBac shRNA expression system (System Biosciences) and transfected into Hepa 1–6 cells. Single clones of stably transfected cells were selected with 3μg/ml puromycin for 2 weeks. Knockdown efficiencies were evaluated by qPCR and western blot analysis on individual clones and the best clones were selected. Target sequences are listed in a table below.

### Cell culture

HEK293A, HEK293T and Hepa1-6 cells were maintained in DMEM medium with 10% FBS and 1x penicillin/streptomycin (100IU and 100ug/ml). HEK293A/T cells also required 1x Non-essential amino acids (Sigma M7145). Primary hepatocytes were isolated and cultured as described [50]. For virus infections, MOI (multiplicity of infeciton) of 100 was used and cells were assayed 3 days after infection.

### Quantitative PCR and expression profiling

RNA was isolated using Trizol (Invitrogen) and cDNA was generated by reverse transcription with RevertAid reverse transcriptase (Fermentas). Relative gene expression was measured using a StepOnePlus machine (Applied Biosystems). Expression profiling was performed using Illumina Mouse Sentrix-6 chips and the data were analysed in Chipster.

### Animal experiments

8–10 week C57BL/6J male mice were purchased from Charles River and maintained with unlimited water and normal chow food in a 12 hour light-dark cycle. After 1 week of adaptation, mice were tail vein-injected with 1x10^11^ viral particles/mouse of either control or knockdown AAV diluted in PBS. After 7 week of infection, mice were subjected to ad libitum feeding, 16 hour fasting or 16 hour fasting + 6 hour refeeding. Food intake for C57BL/6J mice was monitored in an automated metabolic measurement system (TSE Systems).

9 week db/db male mice were also purchased from Charles River and maintained under the same condition as C57BL/6J mice. For virus injection, 2x10^11^ viral particles/mouse were tail-injected. After 5 week of infection, db/db mice were subjected to ad libitum feeding and sacrificed. Mouse body weight and blood glucose levels were monitored regularly. Whole body composition analysis was performed for db/db mice using an Echo MRI system.

For assaying liver PPP2R5C expression in mice on a high-fat versus low-fat diet (S1E Fig), 8-week old male C57BL6/N were fed either a low fat diet (Research Diets Inc. D12450B) or a high fat diet (Research Diets Inc. D12492) for 4 weeks after which they were sacrificed in the fed state and total liver RNA was extracted by Trizol and gene expression was quantified by quantitative RT-PCR.

### Metabolic analyses

Glucose levels in cell culture medium were measured using the Glucose HK assay kit from Sigma (GAHK20-1KT) whereas lactate was measured using the D/L-Lactic acid kit from Roche (11 112 821 035). Non-esterified fatty acids in culture media were first extracted with methanol-chloroform as previously described [51], and then measured with the Free Fatty Acid Fluorometric Assay Kit from Caymen Chemical (700310). Triglycerides were first extracted from cell lysate or tissues with methanol-chloroform as previously described [51] and then digested with lipase (Genzyme, LIPA-70-1461) overnight. The released glycerol was measursed using the Free Glycerol Kit from Sigma (F6428-40ML). Glycogen was extracted from liver in 30% KOH at 95°C for 30 min, and then precipitated in 60% EtOH and re-suspended in water for measurement. Glycogen was first converted into glucose by overnight amyloglucosidase digestion (Sigma 10115) and the resulting glucose was quantified with the Glucose HK kit from Sigma (GAHK20-1KT). Cholesterol was first extracted from tissues with methanol-chloroform as previously described [51], and then measured using the Cholesterol kit from Randox (CH201). Glycolysis rate was measured using the Seahorse glycolysis stress kit on a XF96 analyzer (Seahorse bioscience)

### Glucose tolerance test, insulin sensitivity test and pyruvate tolerance test

C57BL/6J mice 4 weeks after infection with control or knockdown AAV were starved for 6 hours and then injected intraperitoneally with glucose (2g/kg body weight). Blood glucose was measured at 0, 20, 60, 90, 120, and 150 min after injection using a glucose meter from One-Touch (Lifescan). Circulating insulin was measured from 50 μl of blood at 0, 20, and 60 min after glucose injection. For Insulin sensitivity test, C57BL/6J mice with 4 week PPP2R5C HepKD were starved for 6 hours, and then 1IU/kg insulin (Humulin, Eli Lilly) was injected via the tail. Liver samples were collected 10 min after injection. Pyruvate tolerance test was done similarly to the glucose tolerance test. 2g/kg pyruvate was injected intraperitoneally and blood glucose levels were monitored at 0, 20, 40, 60, 90, 120, and 180 minutes after injection.

### Insulin tolerance test

Db/db mice with 4 week of virus infection were subjected to insulin tolerance test. 1.5IU/kg Insulin (Humulin, Eli Lilly) was injected intraperitoneally and blood glucose levels were monitored at 0, 20, 40, 60, 90, 120, 150, 180 and 240 minutes after injection.

### Serum lipoprotein analysis

200μl of pooled serum from 5 or 6 mice was mixed with 100μl PBS and then injected onto a Superose 6 10/300 GL column in an AKTA FPLC purifier for size exclusion chromatography. Separated VLDL, IDL, LDL and HDL were collected in 500μl/fraction and 160 ul and 40 ul of each fraction was used to measure triglyceride and cholesterol levels as described above.

### Serum insulin and ALT measurement

Serum insulin was measured with an ELISA kit from Alpco (80-INSMSU-E01). Serum ALT levels were measured with the Infinity ALT/GPT Reagent (Thermo Scientific TR71121).

### Glucose uptake assay

Stable cell lines with inducible shRNAs or control hepa 1–6 cells were induced with 30**μ**g/ml cumate for 3–4 days and then starved in serum-free DMEM overnight. Then cells were sensitized in KRPH buffer (20 mM HEPES, 5 mM KH2PO4, 1 mM MgSO4, 1 mM CaCl2, 136 mM NaCl, 4.7 mM KCl, pH to 7.4) for 1 hour and treated with 100 μM 2NBDG for 20 min to allow 2NBDG uptake. Uptake was stopped by washing with PBS 3 times. Cells were then trypsinized for 3 min to detach them, and trypsinization was stopped by adding an equal volume of fetal bovine serum. All cells were resuspended and washed in PBS with 2% FBS for 3 times before FACS measurement. 2NBDG intensity was recorded on BD’s FACSCanto II. FACS data were analysed either in FlowJo or R.

For measurement of glucose consumption from medium, Hepa 1–6 cells were infected by adenovirus carrying shRNA targeting all mouse PPP2R5C isoforms (PPP2R5C KD) or a negative-control scramble shRNA (Control KD). After 48h, cells were given fresh DMEM medium. Glucose levels in the medium were quantified using the Glucose HK assay kit from Sigma (GAHK20-1KT) both prior to incubation with the cells, as well as after 24 hours. Glucose consumption was normalized to total cell protein.

### Glycolysis measurement

Hepa 1–6 cells were detached by trypsinization, washed once with 180 μL assay medium (DMEM (sigma D5030) with 143mM NaCl, 2 mM L-Glutamine, pH 7.35 ± 0.05. Adjust pH at day of assay) and incubated with 175 μL assay medium at 37°C for 1 hour in XF96 glycolysis stress kit 96-well plates (Seahorse Bioscience). Then 25 μL of glucose (10 mM in assay medium), oligomycin (2.5 or 1 μM in assay medium) and 2-Deoxyglucose (100 mM in assay medium) each were injected into the plate reservoir and glycolysis rate was recorded on XF96^e^ Extracellular Flux Analyzer from Seahorse Bioscience. All the data collection and analysis was done on XF96 built-in software. Data were normalized to cell number, counted using a Cell Profiler after DAPI staining.

### Fatty acid oxidation assay

Fatty acid beta-oxidation activity in Hepa 1–6 was measured using the Mito Stress kit (Seahorse Bioscience) +/- Etomoxir treatment. Hepa 1–6 cells were replated in XF96 cell culture microplates 24 hours before the experiment and incubated with substrate-limited medium (DMEM with 0.5 mM glucose, 1 mM GlutaMAX, 0.5 mM carnitine, and 1% FBS.). Cells were washed in FAO assay medium (111 mM NaCl, 4.7 mM KCl, 1.25 mM CaCl2, 2 mM MgSO4, 1.2 mM NaH2PO4, 2.5 mM glucose, 0.5 mM carnitine, and 5 mM HEPES pH 7.4) 45 minutes before the experiment. OCR (oxygen consumption rate) measurement was performed by using the Mito Stress kit with 2uM Oligomycin, 2**μ**M FCCP and 0.5**μ**M Retenone/Antimycin A. 400 **μ**M Etomoxir was used to inhibit beta-oxidation activity.

### Euglycemic-hyperinsulinemic clamp

Insulin sensitivity was assessed with the euglycemic-hyperinsulinemic clamp method using a previously described protocol [52]. In brief, after an overnight fast and resting for 30min in supine position, intravenous catheters were inserted into antecubital veins in both arms of the study participants. One was used for the infusion of insulin and glucose, the other was used for frequent blood sampling. After a priming dose of 1.2nmol/m^2^ insulin, infusion with insulin (Actrapid 100 U/ml, Novo Nordisk, Bagsvaerd, Denmark) was started with a constant infusion rate of 0.28nmol/m^2^ body surface per min and continued for at least 120min. After 3min, the variable glucose infusion rate (20% glucose) was added and adjusted during the clamp to maintain the blood glucose at 5.0mmol/l. Bedside blood glucose measurements were carried out every 5min. Glucose infusion rate (GIR) was calculated from the last 45 min of the clamp, in which glucose infusion rate could be kept constant in order to achieve the target plasma glucose concentration of 5.0(± 5%) mmol/l. Therefore, the duration of the clamp varied between individuals (range 120-200min). In premenopausal women, clamp studies were performed during the luteal phase of the menstrual cycle.

### PPP2R5C gene expression analysis in human liver

PPP2R5C mRNA expression was investigated in liver tissue samples obtained from 66 Caucasian men and women (BMI range: 22.7–45.6 kg/m^2^) with (n = 26) or without (n = 40) type 2 diabetes who underwent open abdominal surgery for Roux-en-Y bypass, sleeve gastrectomy, explorative laparotomy or elective cholecystectomy. A small liver biopsy was taken during the surgery, immediately frozen in liquid nitrogen, and stored at −80°C until further preparations. All baseline blood samples were collected between 8 and 10 am after an overnight fast. All study protocols have been approved by the ethics committee of the University of Leipzig (363-10-13122010 and 017-12-230112) in accordance with the principles of the WMA Declaration of Helsinki. All participants gave written informed consent before taking part in the study. Human PPP2R5C mRNA expression was measured by quantitative real-time RT-PCR in a fluorescent temperature cycler using TaqMan assay-on-demand kits (Hs00604899_g1, Applied Biosystems, Darmstadt, Germany), and fluorescence was detected on an ABI PRISM 7000 sequence detector (Applied Biosystems, Darmstadt, Germany). PPP2R5C mRNA expression was calculated relative to the mRNA expression of 18S rRNA (Hs99999901_s1, Applied Biosystems, Darmstadt, Germany).

### PPP2R5C gene expression analysis in human adipose tissue

Adipose tissue samples were obtained from a biobank collection of the University Hospital Joan XXIII (Tarragona, Spain). The Hospitals’ ethics committee approved the study and written informed consent was obtained from all participants. We used paired subcutaneous and visceral adipose tissue samples from healthy and type 2 diabetic patients, matched for age, gender and body mass index (see S6A Fig). All subjects were Caucasian and reported steady body weight for at least 3 months prior to the study. Subjects were scheduled for an elective surgical procedure (cholecystectomy or surgery for abdominal hernia); and had no metabolic diseases other than type 2 diabetes. They had been free of any infections in the month preceding the study. Exclusion criteria were: presence of liver or renal diseases, malignancy, chronic inflammatory disease and pharmacological treatments that may alter the lipid profile. All patients had fasted overnight, for at least 12 h, before surgical procedure. Adipose tissue samples were obtained during the surgical procedure, washed in PBS, immediately frozen in liquid N_2_ and stored at -80°C.

Total RNA was extracted from adipose tissue using the RNeasy lipid tissue midi kit (QIAGEN Science). One microgram of RNA was reverse transcribed with random primers using the reverse transcription system (Applied Biosystems). PPP2R5C (Hs00604899_g1) quantitative gene expression was evaluated with TaqMan low-density arrays (Applied Biosystems; microfluidic cards). Results were calculated using the comparative cycle threshold (Ct) method (2^-ΔΔCt^) and expressed relative to the expression of the housekeeping genes cyclophilin 1A (PPIA).

### Immunoprecipitation, western blotting and antibodies

For immunoprecipitation, cells were lysed in IP buffer (150mM NaCl, 50mM Tris pH7.5, 1% Triton X-100) clarified by centrifugation at 14,000 rpm for 15 minutes, then incubated with primary antibody for 2 hours and then incubated with Protein A-Agarose beads (Roche) for 30 min before washing in IP buffer 3 times. Protein samples for immunoblotting were lysed in 1x Lamelli buffer and subjected to SDS-PAGE with a tris-glycine buffer system. Rabbit antibodies against phospho-GSK3**β**(S9), phospho-S6K(T389), S6K, phospho-AKT(T308), phospho-AKT(T473), AKT, phospho-AMPK**α**(T172), AMPK**α**, AMPK **β**1, phospho-ACC1, ACC1, phospho-TBC1D1(S700), TBC1D1, HSP90, YAP, TSC1, and Rpl26 were from Cell Signaling. PPARα antibody (3B6/PPAR) was purchased from Alexis (Now part of Enzo life sciences). LXR**α** **/** **β** was from Santa Cruz. Mouse antibody anti-SREBP-1 was from BD Biosciences. Anti-HA antibody was from Roche. Anti-FLAG(M2) was from Sigma. Monoclonal mouse anti-Actin was from Developmental Studies Hybridoma Bank. Anti-PPP2R5C was generated by immunizing guinea pigs with NCBI Variant 3 of mouse PPP2R5C.

### PP2A substrate trapping

Potential PPP2R5C substrates were identified by proximity biotinylation using the BioID method described in [33]. PPP2R5C was fused to a mutated biotin ligase (BirA R118G) either to its C terminal (Myc-BirA-PPP2R5C) or N terminal ends (PPP2R5C-BirA-HA). These were co-transfected with or without a catalytic dead version (D85N) of the PP2A catalytic C subunit [34]. After 24 hours of expression, cells were washed with PBS twice and lysed in BioID lysis buffer (50 mM Tris, pH 7.4, 500 mM NaCl, 0.4% SDS, 5 mM EDTA, 1 mM DTT, 2% Triton X-100, and 1x Complete protease inhibitor (Roche)). The lysates were mixed with an equal volume of 50mM Tris (pH 7.4) before incubating with pre-washed streptavidin magnetic beads (Invitrogen MyOne Streptavidin C1) overnight at 4°C. Then the beads were washed twice with 2% SDS, once with BioID wash buffer 1 (0.1% deoxycholate, 1% Triton X-100, 500 mM NaCl, 1 mM EDTA, and 50 mM Hepes, pH 7.5), once with BioID wash buffer 2 (250 mM LiCl, 0.5% NP-40, 0.5% deoxycholate, 1 mM EDTA, and 10 mM Tris, pH 8.1), and twice with wash buffer 3 (50 mM Tris, pH 7.4, and 50 mM NaCl). Finally, all biotinylated proteins were eluted in 1x Laemmli buffer with saturated biotin (around 1mM) at room temperature for 15min and following 15 min boiling at 95°C. Eluted proteins were probed by immunoblotting.

### Proximity ligation assay (PLA)

Mouse PPP2R5C variant 1 and AMPK beta1 were tagged with HA and FLAG tags respectively in pcDNA3 and transfected into Hepa 1–6 cells. Two days after transfection, cells were fixed in 4% PFA and permeabilized in 1x PBX (1xPBS, 0.2% Triton X-100) and stained with anti-HA and anti-FLAG antibodies as per manufacturer instructions (Duolink). Duolink in situ detection reagent red (Duolink) was employed to detect the AMPK beta1-PPP2R5C interaction signal. Nuclei were stained with DAPI.

### Statistical analysis methods

The Student t-test was employed to test the significant difference in various mRNA levels or metabolic phenotypic data among different nutritional statuses, or between control knockdown and PPP2R5C knockdown, or human liver PPP2R5C mRNA levels between healthy control and type 2 diabetic patients. Human adipose tissue PPP2R5C mRNA levels was tested by Mann-Whitney test. Liver triglyceride was by one-way ANCOVA with liver NEFA as covariate. Time course related studies, including GTT, ITT, PTT, seahorse experiments, body weight or glucose changing profiles, were analysed by 2-way ANOVA with time and PPP2R5C knockdown as factors. The difference at individual time points for GTT was also tested by Wilcoxon signed-rank test. Correlation analysis between human liver PPP2R5C mRNA levels and glucose infusion rate was determined by Pearson’s correlation method. All data analysis was performed in R 3.0.1.

Sequences of oligos, shRNAs and miRNAs used are provided in S1 Table.

### Ethics statement

For PPP2R5C expression in human liver, all study protocols were approved by the ethics committee of the University of Leipzig (363-10-13122010 and 017-12-230112) in accordance with the principles of the WMA Declaration of Helsinki. Adipose tissue samples were obtained from a biobank adipose tissue collection (internal approval code: 54c/2009) of the University Hospital Joan XXIII (Tarragona, Spain), according to the biomedical research law 14/2007 from Spain, and with written informed consent from participants. Animal experiments were conducted according to local, national, and EU ethical guidelines and approved by local regulatory authorities (Regierungspräsidium Karlsruhe).

## Supporting Information

S1 Fig Supporting data for main Fig 1. **(A)** PPP2R5C protein levels are not changed upon nutritional status change. PPP2R5C protein levels were detected in liver sample of mice under various feeding conditions as described in Fig 2. **(B)** PPP2R5C mRNA levels are down-regulated by insulin and hFGF19. Mouse primary hepatocytes were stimulated with 100nM insulin or 10nM recombinant human FGF19 (human ortholog of moues FGF15) for 3 hours. (n = 3). **(C)** PPP2R5C mRNA levels are responsive to PPAR alpha activation. Mouse primary hepatocytes were stimulated with PPAR alpha agonist wy14643 at the indicated concentrations for 24 hours. CPT1A mRNA levels were used as positive control for a bona fide PPAR alpha target. (n = 3). **(D)** Leptin treatment (24h) of primary hepatocytes does not increase PPP2R5C expression. **(E)** Hepatic PPP2R5C expression is mildly increased in C57BL6/N mice fed a high-fat diet (HFD) for 4 weeks. (p-value HFD vs LFD = 0.08, n = 8). Error bars: std. dev. *p-value<0.05 and **p-value<0.01 by student t-test (B-C).
(TIF)

S2 Fig PPP2R5C HepKD mice have improved insulin sensitivity. **(A-A’)** PPP2R5C knockdown efficiency in mouse liver in vivo. Knockdown was performed by tail injecting an adeno-associated virus bearing a miRNA targeting PPP2R5C, or a non-targeting negative control, as in Fig 2. Seven weeks post injection, PPP2R5C protein levels were detected using a self-made antibody (A) and mRNA levels were quantified by Q-RT-PCR (A’). (n = 5) **(B-C)** Knockdown of liver PPP2R5C has no significant effect on serum ALT levels (B) and body weight (C). **(D)** Insulin levels for the glucose tolerance test shown in Fig 2C are not elevated in PPP2R5C HepKD mice compared to controls. (n = 12) **(E)** PPP2R5C knockdown efficiency in Hepa 1–6 was tested by infecting Hepa 1–6 cells with adenovirus bearing a shRNA targeting PPP2R5C, or a non-targeting negative control. PPP2R5C protein levels were detected 3 days after infection using the same antibody as in (A). **(F)** PPP2R5C knockdown efficiency in Hepa 1–6 cells using 2 independent inducible shRNA was tested by generating stably-transfected Hepa 1–6 cell lines and detecting PPP2R5C protein levels 3 days after induction of shRNA with varying concentrations of inducer (cumate). **(G)** Pyruvate tolerance test (PTT) shows no change in gluconeogenesis activity after PPP2R5C HepKD in C57BL/6 mice. 2g/kg pyruvate injected intraperitoneally (n = 6). The ascending part of the graph represents gluconeogenesis caused by pyruvate injection. The descending part of the graph represents glucose clearance (similar to a glucose tolerance test). **(H)** Liver gluconeogenesis markers, PCK1, G6PC, and PPARGC1A, are not dramatically changed in all feeding regimes upon PPP2R5C knockdown (n = 5 or 6). Liver samples were the same as in Fig 2. Error bars: std. dev. *p-value<0.05, **p-value<0.01 by student t-test (A’,D,H).
(TIF)

S3 Fig PPP2R5C KD promotes de novo lipogenesis. **(A)** Food intake is not changed upon PPP2R5C knockdown. PPP2R5C was knockdown by adeno-associated virus as in Fig 2 for two week, and then food intake was monitored in TSE Systems for 1 week (n = 12). **(B)** Short term knockdown of PPP2R5C (2 weeks post tail-injection of miRNA-bearing adeno-associated virus) significantly increases liver triglyceride levels in the fed state. (n = 5) **(C)** Knockdown of liver PPP2R5C has no significant effect on serum ketone body levels. (n = 5–6) **(D)** PPP2R5C KD in Hepa 1–6 mildly increases beta-oxidation activity. The effects of PPP2R5C KD and time on the OCR rate profile were tested by two-way ANOVA. p-value for PPP2R5C KD under basal (BSA treated) and palmitate stimulation were 0.03 and <2x10^-16^ respectively. PPP2R5C was knocked down as shown in Fig 2F. **(E)** Depletion of non-esterified free fatty acids (NEFA) from the medium of Hepa 1–6 cells was not changed by PPP2R5C knockdown. Knockdown conditions were the same as in main Fig 3. NEFA consumption was measured during a 72 hour time window after PPP2R5C knockdown (n = 3). **(F)** PPP2R5C KD in Hepa 1–6 cells does not lead to increased triglyceride secretion into the medium. PPP2R5C was knocked down as shown in Fig 2F. (n = 3) **(G-H)** Knockdown of liver PPP2R5C decreases cholesterol storage in liver (H), and increases VLDL secretion upon fasting or refeeding (G). (n = 5–6) **(I)** Circulating free fatty acid levels are not significantly different in serum of PPP2R5C HepKD animals compared to control animals upon fasting (n = 5–6). Error bars: std. dev. *p-value<0.05, **p-value<0.01 by student t-test (B,H).
(TIF)

S4 Fig PPP2R5C interacts with AMPK beta 1. **(A)** BioID identifies AMPK beta 1 as a PPP2R5C interacting protein. Proteins interacting with PPP2R5C in vivo in Hepa 1–6 cells were biotinylated and purified as in Fig 4A, and probed by immunoblotting. RpL26, SREBP1, TSC1, YAP, and HSP90 were included as negative control proteins which were not detected in the pulldowns. **(B)** Validation of AMPK beta 1 interaction with PPP2R5C using Proximity Ligation Assay (PLA) as an independent method. HA-tagged mouse PPP2R5C variant 1 and FLAG-tagged mouse AMPK beta 1 were co-overexpressed in Hepa 1–6 and the interaction was detected by PLA, seens as red puncta. Nuclei were stained with DAPI.
(TIF)

S5 Fig PPAR alpha targets in liver does not increase during fasting upon PPP2R5C HepKD. **(A)** Expression of PPAR alpha target genes is not increased upon PPP2R5C knockdown in mouse liver in vivo. PPP2R5C was knocked-down in vivo using adeno-associated virus as in Fig 2. PPAR alpha target genes quantified by Q-RT-PCR, normalized to TBP. **(B)** Expression of LXR target genes, SREBP1c and SREBP1a, is increased upon PPP2R5C knockdown in mouse liver in vivo. LXRa/b, SREBP1a/c mRNA levels were quantified by Q-RT-PCR normalized to TBP. Error bars: std. dev. *p-value<0.05, **p-value<0.01, ***p-value<0.001 by student t-test (A-B).
(TIF)

S6 Fig PPP2R5C expression is elevated in subcutaneous adipose tissue of diabetic patients. **(A)** Characteristics of the population used in the study of PPP2R5C expression in adipose tissue of diabetic patients. **(B)** Human PPP2R5C mRNA levels are significantly elevated in Subcutaneous adipose tissue (SAT) of type 2 diabetic patients (n = 11) when compared with healthy counterparts (n = 36). Mann-Whitney test: p = 0.042.
(TIF)

S1 Table Genes dysregulated upon PPP2R5C knockdown in cell culture. (XLSX)

S1 Materials Sequences of primers, shRNAs and miRNAs used. (PDF)
