# Supplementary figures and images for: PPP2R5C Couples Hepatic Glucose and Lipid Homeostasis
Source: PLoS Genet. 2015 Oct 6;11(10):e1005561. doi: 10.1371/journal.pgen.1005561 (PMC4595073; doi:10.1371/journal.pgen.1005561)

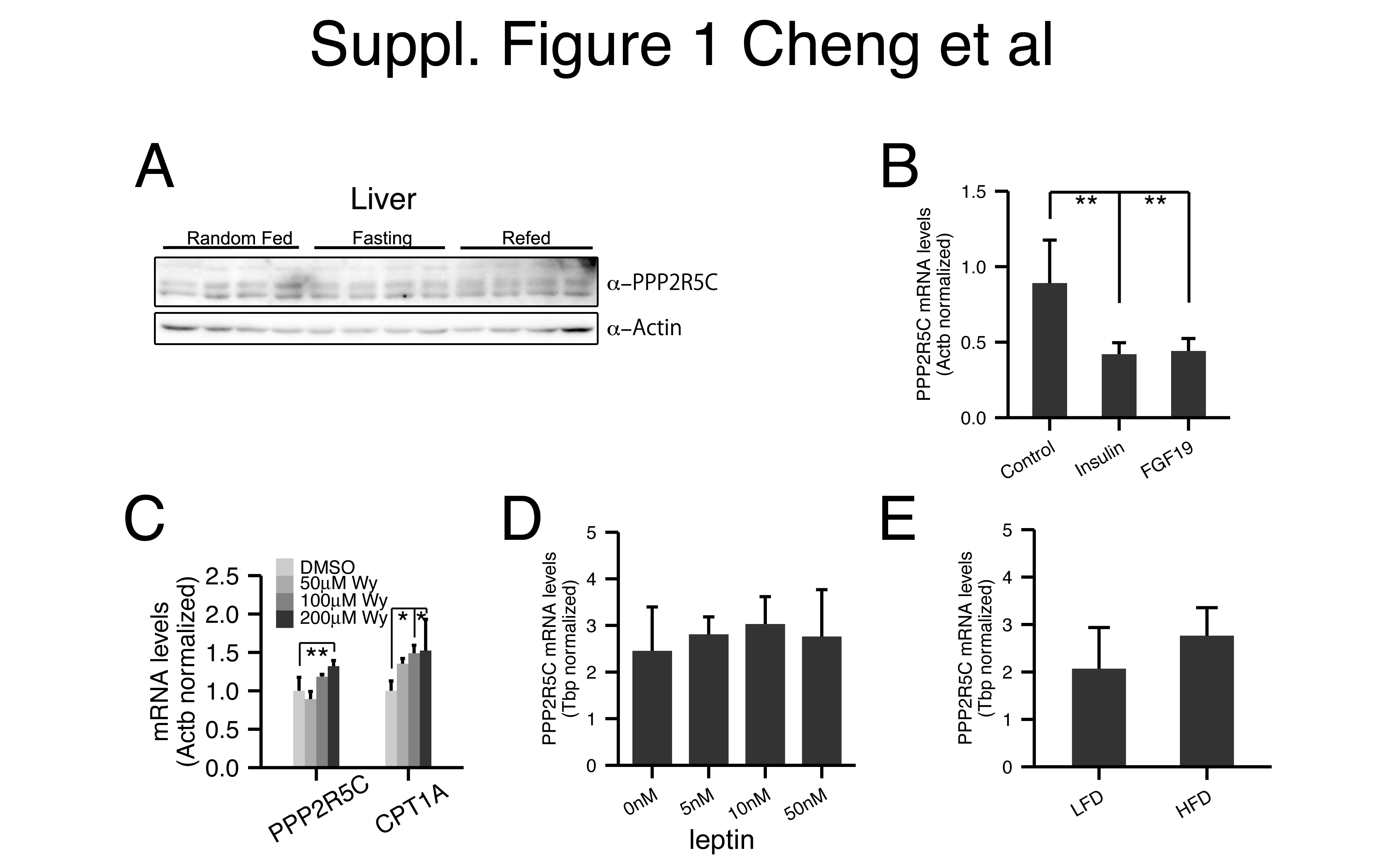

Supplement: S1 Fig — (A) PPP2R5C protein levels are not changed upon nutritional status change. PPP2R5C protein levels were detected in liver sample of mice under various feeding conditions as described in Fig 2. (B) PPP2R5C mRNA levels are down-regulated by insulin and hFGF19. Mouse primary hepatocytes were stimulated with 100nM insulin or 10nM recombinant human FGF19 (human ortholog of moues FGF15) for 3 hours. (n = 3). (C) PPP2R5C mRNA levels are responsive to PPAR alpha activation. Mouse primary hepatocytes were stimulated with PPAR alpha agonist wy14643 at the indicated concentrations for 24 hours. CPT1A mRNA levels were used as positive control for a bona fide PPAR alpha target. (n = 3). (D) Leptin treatment (24h) of primary hepatocytes does not increase PPP2R5C expression. (E) Hepatic PPP2R5C expression is mildly increased in C57BL6/N mice fed a high-fat diet (HFD) for 4 weeks. (p-value HFD vs LFD = 0.08, n = 8). Error bars: std. dev. *p-value<0.05 and **p-value<0.01 by student t-test (B-C). (TIF) [file pgen.1005561.s001.tif]

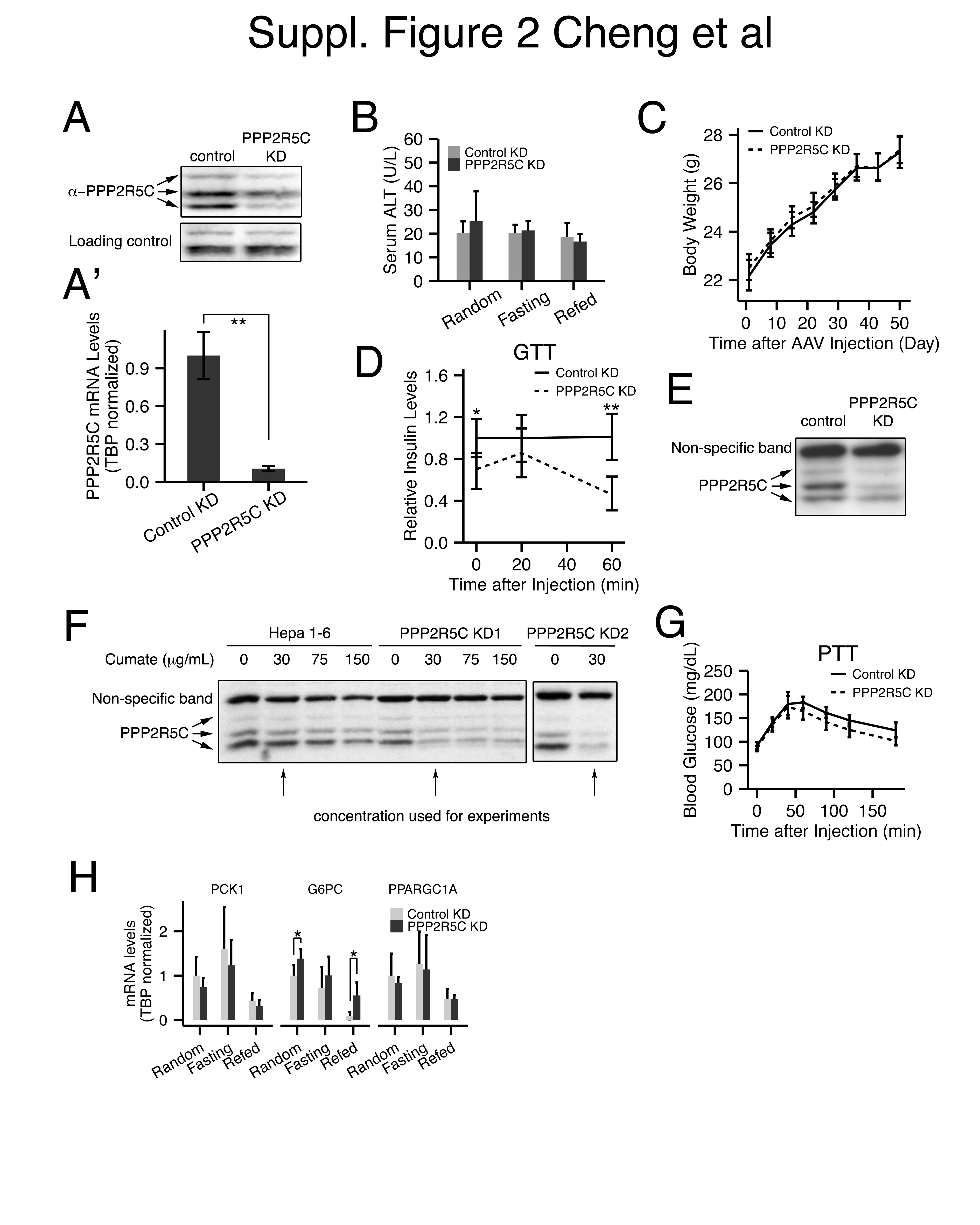

Supplement: S2 Fig — (A-A’) PPP2R5C knockdown efficiency in mouse liver in vivo. Knockdown was performed by tail injecting an adeno-associated virus bearing a miRNA targeting PPP2R5C, or a non-targeting negative control, as in Fig 2. Seven weeks post injection, PPP2R5C protein levels were detected using a self-made antibody (A) and mRNA levels were quantified by Q-RT-PCR (A’). (n = 5) (B-C) Knockdown of liver PPP2R5C has no significant effect on serum ALT levels (B) and body weight (C). (D) Insulin levels for the glucose tolerance test shown in Fig 2C are not elevated in PPP2R5C HepKD mice compared to controls. (n = 12) (E) PPP2R5C knockdown efficiency in Hepa 1–6 was tested by infecting Hepa 1–6 cells with adenovirus bearing a shRNA targeting PPP2R5C, or a non-targeting negative control. PPP2R5C protein levels were detected 3 days after infection using the same antibody as in (A). (F) PPP2R5C knockdown efficiency in Hepa 1–6 cells using 2 independent inducible shRNA was tested by generating stably-transfected Hepa 1–6 cell lines and detecting PPP2R5C protein levels 3 days after induction of shRNA with varying concentrations of inducer (cumate). (G) Pyruvate tolerance test (PTT) shows no change in gluconeogenesis activity after PPP2R5C HepKD in C57BL/6 mice. 2g/kg pyruvate injected intraperitoneally (n = 6). The ascending part of the graph represents gluconeogenesis caused by pyruvate injection. The descending part of the graph represents glucose clearance (similar to a glucose tolerance test). (H) Liver gluconeogenesis markers, PCK1, G6PC, and PPARGC1A, are not dramatically changed in all feeding regimes upon PPP2R5C knockdown (n = 5 or 6). Liver samples were the same as in Fig 2. Error bars: std. dev. *p-value<0.05, **p-value<0.01 by student t-test (A’,D,H). (TIF) [file pgen.1005561.s002.tif]

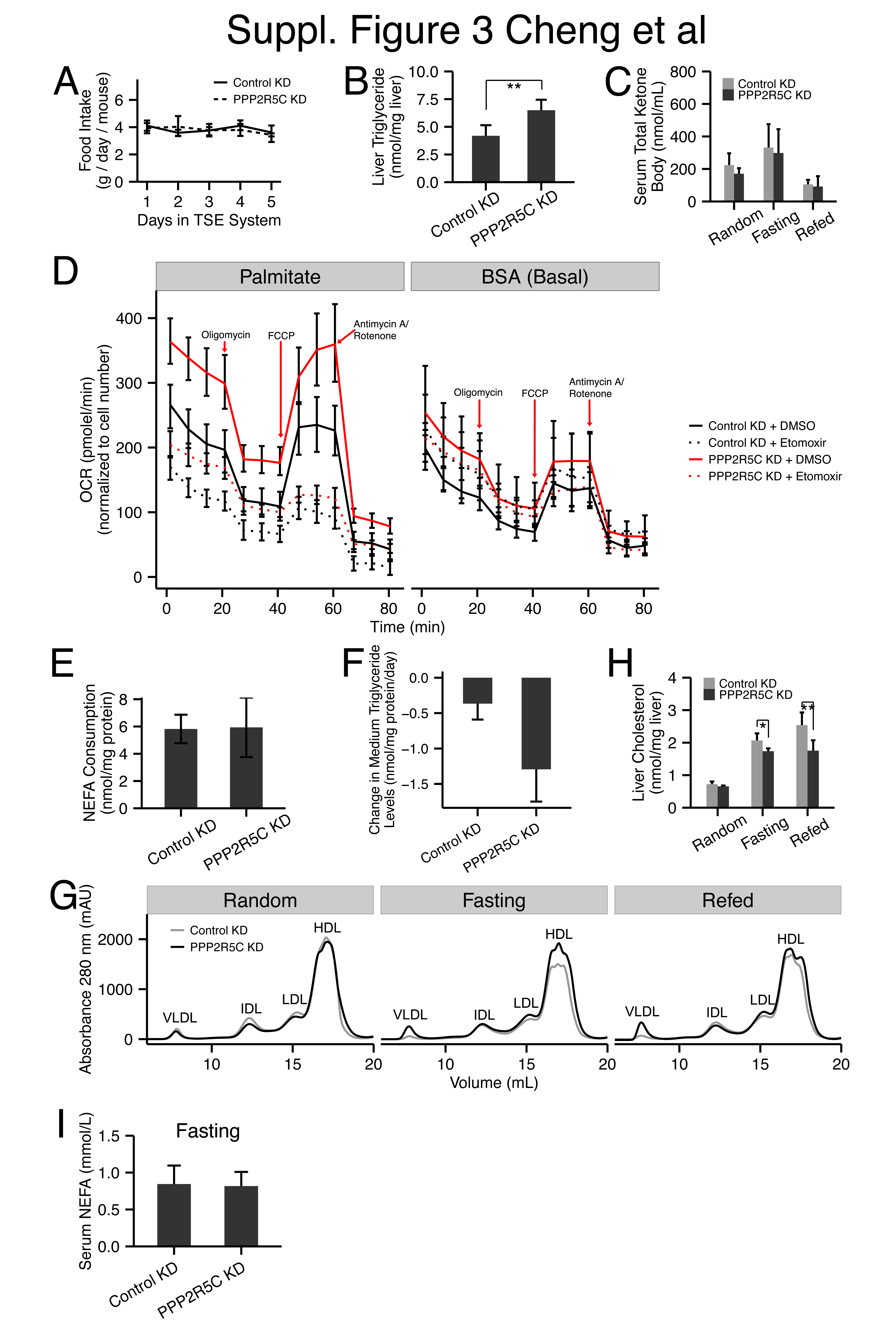

Supplement: S3 Fig — (A) Food intake is not changed upon PPP2R5C knockdown. PPP2R5C was knockdown by adeno-associated virus as in Fig 2 for two week, and then food intake was monitored in TSE Systems for 1 week (n = 12). (B) Short term knockdown of PPP2R5C (2 weeks post tail-injection of miRNA-bearing adeno-associated virus) significantly increases liver triglyceride levels in the fed state. (n = 5) (C) Knockdown of liver PPP2R5C has no significant effect on serum ketone body levels. (n = 5–6) (D) PPP2R5C KD in Hepa 1–6 mildly increases beta-oxidation activity. The effects of PPP2R5C KD and time on the OCR rate profile were tested by two-way ANOVA. p-value for PPP2R5C KD under basal (BSA treated) and palmitate stimulation were 0.03 and <2x10^-16^ respectively. PPP2R5C was knocked down as shown in Fig 2F. (E) Depletion of non-esterified free fatty acids (NEFA) from the medium of Hepa 1–6 cells was not changed by PPP2R5C knockdown. Knockdown conditions were the same as in main Fig 3. NEFA consumption was measured during a 72 hour time window after PPP2R5C knockdown (n = 3). (F) PPP2R5C KD in Hepa 1–6 cells does not lead to increased triglyceride secretion into the medium. PPP2R5C was knocked down as shown in Fig 2F. (n = 3) (G-H) Knockdown of liver PPP2R5C decreases cholesterol storage in liver (H), and increases VLDL secretion upon fasting or refeeding (G). (n = 5–6) (I) Circulating free fatty acid levels are not significantly different in serum of PPP2R5C HepKD animals compared to control animals upon fasting (n = 5–6). Error bars: std. dev. *p-value<0.05, **p-value<0.01 by student t-test (B,H). (TIF) [file pgen.1005561.s003.tif]

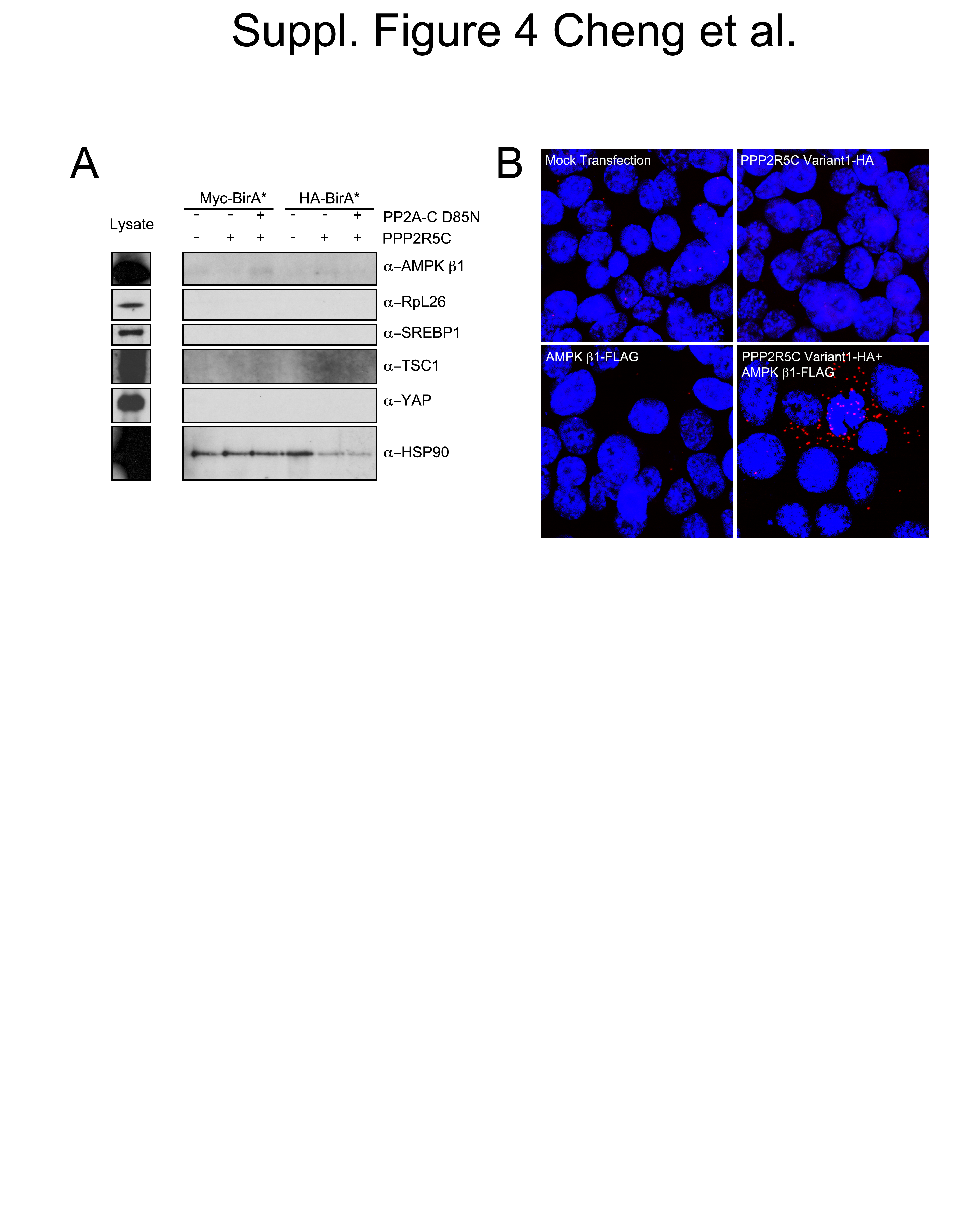

Supplement: S4 Fig — (A) BioID identifies AMPK beta 1 as a PPP2R5C interacting protein. Proteins interacting with PPP2R5C in vivo in Hepa 1–6 cells were biotinylated and purified as in Fig 4A, and probed by immunoblotting. RpL26, SREBP1, TSC1, YAP, and HSP90 were included as negative control proteins which were not detected in the pulldowns. (B) Validation of AMPK beta 1 interaction with PPP2R5C using Proximity Ligation Assay (PLA) as an independent method. HA-tagged mouse PPP2R5C variant 1 and FLAG-tagged mouse AMPK beta 1 were co-overexpressed in Hepa 1–6 and the interaction was detected by PLA, seens as red puncta. Nuclei were stained with DAPI. (TIF) [file pgen.1005561.s004.tif]

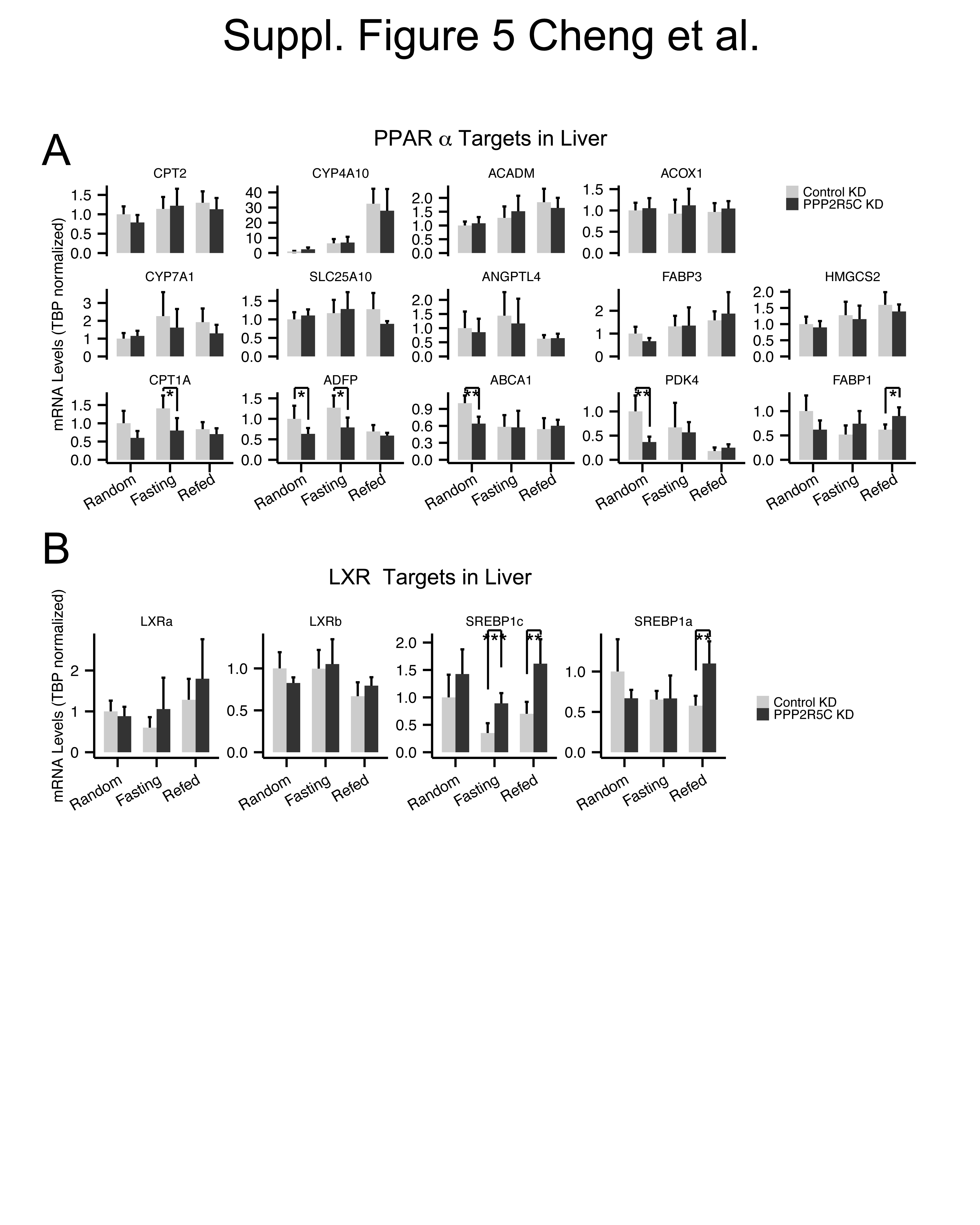

Supplement: S5 Fig — (A) Expression of PPAR alpha target genes is not increased upon PPP2R5C knockdown in mouse liver in vivo. PPP2R5C was knocked-down in vivo using adeno-associated virus as in Fig 2. PPAR alpha target genes quantified by Q-RT-PCR, normalized to TBP. (B) Expression of LXR target genes, SREBP1c and SREBP1a, is increased upon PPP2R5C knockdown in mouse liver in vivo. LXRa/b, SREBP1a/c mRNA levels were quantified by Q-RT-PCR normalized to TBP. Error bars: std. dev. *p-value<0.05, **p-value<0.01, ***p-value<0.001 by student t-test (A-B). (TIF) [file pgen.1005561.s005.tif]

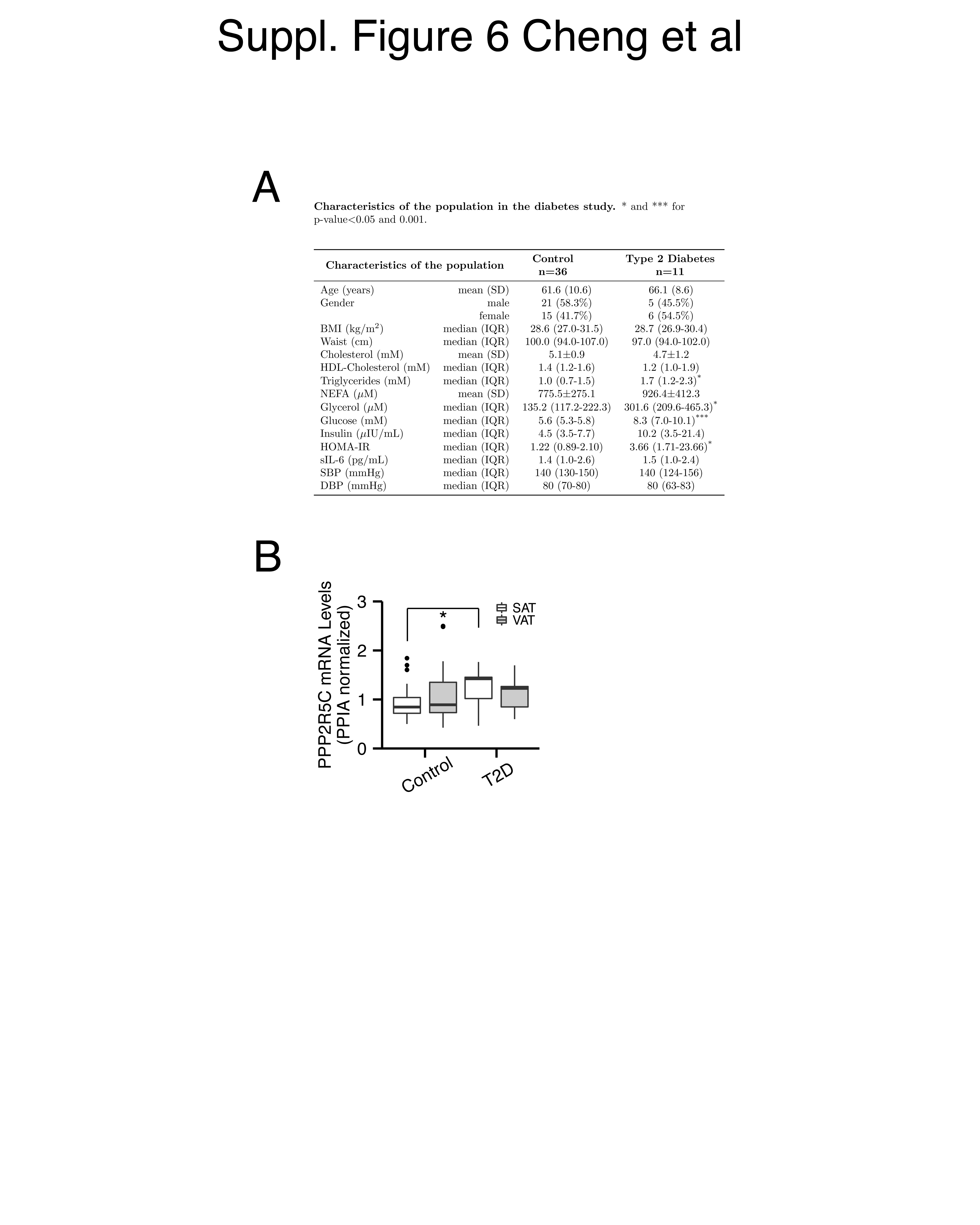

Supplement: S6 Fig — (A) Characteristics of the population used in the study of PPP2R5C expression in adipose tissue of diabetic patients. (B) Human PPP2R5C mRNA levels are significantly elevated in Subcutaneous adipose tissue (SAT) of type 2 diabetic patients (n = 11) when compared with healthy counterparts (n = 36). Mann-Whitney test: p = 0.042. (TIF) [file pgen.1005561.s006.tif]
