# Supplementary material for: PPP2R5C Couples Hepatic Glucose and Lipid Homeostasis
Source: PLoS Genet. 2015 Oct 6;11(10):e1005561. doi: 10.1371/journal.pgen.1005561 (PMC4595073; doi:10.1371/journal.pgen.1005561)
Supplement: S1 Materials — (PDF) [file pgen.1005561.s008.pdf]

Supplemental Material for

"PPP2R5C couples hepatic glucose and lipid homeostasis"

Cheng et al.

1. Quantitative RT-PCR Primer List

Oligo No.	Primer sequence	Purpose
OAT1638	CGTCGTCCCTGTGAAGAA	Forward primer for quantitative RT-PCR mPPP2R5C variant 4
OAT1639	GATCCGCAGGAGGAACAT	Reverse primer for quantitative RT-PCR mPPP2R5C variant 4
OYC175	AGTTTGTATTGCAGCTTCTA	Forward primer for quantitative RT-PCR mPPP2R5C
OYC176	TCCAGTAACTCCGCTATG	Reverse primer for quantitative RT-PCR mPPP2R5C
OYC124	AGGAGTATGGGCTTCATTGGGCA	Forward primer for quantitative RT-PCR mAcly
OYC125	TCCCAGGGGTGACGATACAGCC	Reverse primer for quantitative RT-PCR mAcly
OYC185	CCTGTGCTACCTTCTCTCTA	Forward primer for quantitative RT-PCR mGPAT
OYC186	CTTCCTGGTCATCTTGCTCT	Reverse primer for quantitative RT-PCR mGPAT
OYC197	CACACATCACTTAGCCAAC	Forward primer for quantitative RT-PCR mHMGCS1
OYC198	GTCCTTCTGTGTTTTTCATC	Reverse primer for quantitative RT-PCR mHMGCS1
OYC199	ATCTTCTTCCCTATTGCACT	Forward primer for quantitative RT-PCR mLDLR
OYC200	TGGGTTGTCAAAGTTTATGC	Reverse primer for quantitative RT-PCR mLDLR
OYC209	AGAAAGGTGGCAGGAGATCG	Forward primer for quantitative RT-PCR mDGAT2
OYC210	GTCAGCAGGTTGTGTGTCTT	Reverse primer for quantitative RT-PCR mDGAT2
OYC213	ATGACCAGACTTCCTCCAAC	Forward primer for quantitative RT-PCR mSlc25a1
OYC214	GTATGTTCCCTTTAGCCCTT	Reverse primer for quantitative RT-PCR mSlc25a1
OYC233	AGTTCCATTGACAAGGCCAT	Forward primer for quantitative RT-PCR mSREBP1
OYC234	TACCGTGAGCTACCTGGACT	Reverse primer for quantitative RT-PCR mSREBP1
OYC395	CCATTTCCACCATGATTAAGGGTCT	Forward primer for quantitative RT-PCR mLDHa
OYC396	CGAGATTCCATTTTGTCCCAGGATA	Reverse primer for quantitative RT-

		PCR mLDHa
OYC397	CAGATCTCTCAGCCCGCCAA	Forward primer for quantitative RT-PCR mNDRG1
OYC398	GGCGAGTCATGCTGGCAGAA	Reverse primer for quantitative RT-PCR mNDRG1
OYC399	ACAAGCTTCATCCTCACTTTGCC	Forward primer for quantitative RT-PCR mHK2
OYC400	GGAAGGACACGTCACATTTCGGA	Reverse primer for quantitative RT-PCR mHK2
OYC110	ATGGATGTTGGCAAGGCCCGA	Forward primer for quantitative RT-PCR mPKM2
OYC111	AGGCACTACACGCATGGTGTTGG	Reverse primer for quantitative RT-PCR mPKM2
OYC447	GTACCACCGCCAGTTGTTTG	Forward primer for quantitative RT-PCR mPklr
OYC448	AGGTCGGTAGCGAGACAGAA	Reverse primer for quantitative RT-PCR mPklr
OYC449	CCTGTGGATGCAAGGGTCTC	Forward primer for quantitative RT-PCR mTxnip
OYC450	AGCTCGAAGCCGAACTTGTA	Reverse primer for quantitative RT-PCR mTxnip
OYC451	CGATCTATCCGTCGGTGGTC	Forward primer for quantitative RT-PCR mAcaca
OYC452	TGTTGTTGTTGGGTCCTCCA	Reverse primer for quantitative RT-PCR mAcaca
OYC453	GAAGATCGCCCCACTGAAAG	Forward primer for quantitative RT-PCR mMttp
OYC454	GCCAACACGTCTAGCCAGTAG	Reverse primer for quantitative RT-PCR mMttp
OYC455	GGCTCTGACGATGGGGAAC	Forward primer for quantitative RT-PCR mGlut4
OYC456	GCCACGTTGCATTGTAGCTC	Reverse primer for quantitative RT-PCR mGlut4
OYC457	CAGAGTCCCCGCAGGATACA	Forward primer for quantitative RT-PCR mChREBP
OYC458	CTGATAGTCGCCGCTCACTG	Reverse primer for quantitative RT-PCR mChREBP
OYC459	GAGTGTCCCTCCCAAAGAGC	Forward primer for quantitative RT-PCR mG0s2
OYC460	GGATCAGCTCCTGCACACTT	Reverse primer for quantitative RT-PCR mG0s2
OYC461	GCTCTTTCCCTCGCTCATCA	Forward primer for quantitative RT-PCR mGckr
OYC462	TGAGAACCTTGTATTCAACACCC	Reverse primer for quantitative RT-PCR mGckr
OYC427	CAAGTGTCCACCAACAAGCG	Forward primer for quantitative RT-PCR mFasn
OYC428	GGAGCGCAGGATAGACTCAC	Reverse primer for quantitative RT-PCR mFasn

OYC415	CACCTGGAGATCAGGGAGGA	Forward primer for quantitative RT-PCR mFgf21
OYC416	GCAGGCCTCAGGATCAAAGT	Reverse primer for quantitative RT-PCR mFgf21
OYC413	CAGAAATCCCTGGCTCGGTT	Forward primer for quantitative RT-PCR mHmgcs2
OYC414	TTGAACATGTCCAGGGAGGC	Reverse primer for quantitative RT-PCR mHmgcs2
OYC417	GGACTCCGCTCGCTCATT	Forward primer for quantitative RT-PCR mCpt1a
OYC418	GAGATCGATGCCATCAGGGG	Reverse primer for quantitative RT-PCR mCpt1a
OYC419	GACTATTCGCCCAGCTTCCA	Forward primer for quantitative RT-PCR mCpt2
OYC420	GCTGCCAGATACCGTAGAGC	Reverse primer for quantitative RT-PCR mCpt2
OYC421	TCAAGATCGCAATGGGTGCT	Forward primer for quantitative RT-PCR mAcadm
OYC422	GCTCCACTAGCAGCTTTCCA	Reverse primer for quantitative RT-PCR mAcadm
OYC423	GAATTTGGCATCGCAGACCC	Forward primer for quantitative RT-PCR mAcox1
OYC424	ACGGGTGCATCCATTTCTCC	Reverse primer for quantitative RT-PCR mAcox1
OYC425	CCAAGTGCCTTTCCTAGATG	Forward primer for quantitative RT-PCR mCyp4a10
OYC426	GAACCATGGCTGTCCATTCA	Reverse primer for quantitative RT-PCR mCyp4a10
OYC431	GGAAGGACATCAAGGGGGTG	Forward primer for quantitative RT-PCR mFabp1
OYC432	TCACCTTCCAGCTTGACGAC	Reverse primer for quantitative RT-PCR mFabp1
OYC433	GTGACAGCAGATGACCGGAA	Forward primer for quantitative RT-PCR mFabp3
OYC434	CTCACCACACTGCCATGAGT	Reverse primer for quantitative RT-PCR mFabp3
OYC435	GGACGTGCTCAAGTCTCGAT	Forward primer for quantitative RT-PCR mSlc25a20
OYC436	CGCGGATCATGACTGCATTG	Reverse primer for quantitative RT-PCR mSlc25a20
OYC437	GTGGTGATCTGGACCGTGC	Forward primer for quantitative RT-PCR mADFP/Plin2
OYC438	ACTGACATAAGCGGAGGACAC	Reverse primer for quantitative RT-PCR mADFP/Plin2
OYC439	GGGACTGCCAGGAACTCTTC	Forward primer for quantitative RT-PCR mAngptl4

OYC440	GAAGTCCACAGAGCCGTTCA	Reverse primer for quantitative RT-PCR mAngptl4
OYC441	CTGGGGGATTGCTGTGGTAG	Forward primer for quantitative RT-PCR mCyp7a1
OYC442	GCACAGCCCAGGTATGGAAT	Reverse primer for quantitative RT-PCR mCyp7a1
OYC443	CGACCATGAAAGTGACACGC	Forward primer for quantitative RT-PCR mAbca1
OYC444	AGCACATAGGTCAGCTCGTG	Reverse primer for quantitative RT-PCR mAbca1
OYC445	GAGCTGTTCTCCCGCTACAG	Forward primer for quantitative RT-PCR mPdk4
OYC446	CGGTCAGGCAGGATGTCAAT	Reverse primer for quantitative RT-PCR mPdk4
OYC409	GGGTGGAAGGTCGAATGTGT	Forward primer for quantitative RT-PCR mPck1
OYC410	AGCCCTTAAGTTGCCTTGGG	Reverse primer for quantitative RT-PCR mPck1
OYC411	CAGTGGTCGGAGACTGGTTC	Forward primer for quantitative RT-PCR mG6pc
OYC412	GTCCAGGACCCACCAATACG	Reverse primer for quantitative RT-PCR mG6pc
OYC463	ATTCGGGAGCTGGATGGCTT	Forward primer for quantitative RT-PCR mPpargc1a
OYC464	CCGATTGGTCGCTACACCAC	Reverse primer for quantitative RT-PCR mPpargc1a

shRNAs/miRNAs list

**shR/miRs**	**Target sequence**	**Name in Figures**
shRNA	CGTGCTTACATCAGGAAACA	PPP2R5C KD/KD1
shRNA	TCAGAGTTTGTGAAGATCATG	PPP2R5C KD2
miRNA	AGACAATACACGGCTTGATAT	PPP2R5C KD
